# Microbial succession in an inflated lunar/Mars analog habitat during a 30-day human occupation

**DOI:** 10.1186/s40168-016-0167-0

**Published:** 2016-06-02

**Authors:** Teresa Mayer, Adriana Blachowicz, Alexander J. Probst, Parag Vaishampayan, Aleksandra Checinska, Tiffany Swarmer, Pablo de Leon, Kasthuri Venkateswaran

**Affiliations:** Biotechnology and Planetary Protection Group, Jet Propulsion Laboratory, California Institute of Technology, Pasadena, CA USA; Department of Earth and Planetary Sciences, University of California, Berkeley, Berkeley, CA USA; Department of Space Studies, University of North Dakota, Grand Forks, ND 58202 USA

**Keywords:** Closed habitat, Surface, Bacteriome, Microbial succession

## Abstract

**Background:**

For potential future human missions to the Moon or Mars and sustained presence in the International Space Station, a safe enclosed habitat environment for astronauts is required. Potential microbial contamination of closed habitats presents a risk for crewmembers due to reduced human immune response during long-term confinement. To make future habitat designs safer for crewmembers, lessons learned from characterizing analogous habitats is very critical. One of the key issues is that how human presence influences the accumulation of microorganisms in the closed habitat.

**Results:**

Molecular technologies, along with traditional microbiological methods, were utilized to catalog microbial succession during a 30-day human occupation of a simulated inflatable lunar/Mars habitat. Surface samples were collected at different time points to capture the complete spectrum of viable and potential opportunistic pathogenic bacterial population. Traditional cultivation, propidium monoazide (PMA)–quantitative polymerase chain reaction (qPCR), and adenosine triphosphate (ATP) assays were employed to estimate the cultivable, viable, and metabolically active microbial population, respectively. Next-generation sequencing was used to elucidate the microbial dynamics and community profiles at different locations of the habitat during varying time points. Statistical analyses confirm that occupation time has a strong influence on bacterial community profiles. The Day 0 samples (before human occupation) have a very different microbial diversity compared to the later three time points. Members of *Proteobacteria* (esp. *Oxalobacteraceae* and *Caulobacteraceae*) and *Firmicutes* (esp. *Bacillaceae*) were most abundant before human occupation (Day 0), while other members of *Firmicutes* (*Clostridiales*) and *Actinobacteria* (esp. *Corynebacteriaceae*) were abundant during the 30-day occupation. Treatment of samples with PMA (a DNA-intercalating dye for selective detection of viable microbial population) had a significant effect on the microbial diversity compared to non-PMA-treated samples.

**Conclusions:**

Statistical analyses revealed a significant difference in community structure of samples over time, particularly of the bacteriomes existing before human occupation of the habitat (Day 0 sampling) and after occupation (Day 13, Day 20, and Day 30 samplings). *Actinobacteria* (mainly *Corynebacteriaceae*) and *Firmicutes* (mainly *Clostridiales Incertae Sedis XI* and *Staphylococcaceae*) were shown to increase over the occupation time period. The results of this study revealed a strong relationship between human presence and succession of microbial diversity in a closed habitat. Consequently, it is necessary to develop methods and tools for effective maintenance of a closed system to enable safe human habitation in enclosed environments on Earth and beyond.

**Electronic supplementary material:**

The online version of this article (doi:10.1186/s40168-016-0167-0) contains supplementary material, which is available to authorized users.

## Background

In the industrialized world, humans are spending the majority of their lives indoors—some up to 90 % of their time [1, 2]. Built environments are complex ecosystems known to have specific microbiomes [3]. Bio-contamination of these controlled and enclosed environments can present health risks to inhabitants [4–7]. An airborne microbial biodiversity investigation of Halley Station, an isolated scientific research station in continental Antarctica, was conducted to discern the potential source of microbial population and determined no significant patterns in the aerial biodiversity between the austral summer and austral winter [8]. This investigation, however, did not examine the succession of the microbial population for a defined time period. Subsequently, a 1-year investigation of the environmental airborne bacterial population was conducted in the human occupied Concordia Research Station based on conventional cultivation assays [9]. The airborne cultivable bacterial density of the Concordia Research Station was low (<1.0 × 10^3^ CFU/m^3^), and bacterial contamination was found to increase over time during confinement but diminish after reopening of the station. The predominant cultivable bacterial genera were related to humans (*Staphylococcus* sp. and *Bacillus* sp.), and environmental species, such as *Sphingomonas paucimobilis*, were also found in the air along with a few opportunistic pathogens.

An air sampling study at Providence Milwaukie Hospital revealed that ventilation (air and airflow rates) influenced the microbial community composition. For this study, outdoor and indoor air samples were collected from mechanically ventilated and “naturally” ventilated rooms of the hospital. The relative abundance of bacteria closely related to human pathogens was higher indoors than outdoors and in rooms with lower airflow rates [10]. This study suggests that reducing the airflow rate with the outdoor environment to a minimum allowed the existence of more pathogenic microorganisms. This is an important factor to consider during the habitat design for space missions. In the closed built environments used for space missions, it is not possible to have a constant air flow from the surrounding environment. Therefore, it is very important to understand the ecology of the microbiome in built and closed environments to maximize the health and performance of crewmembers [11].

Similarly, examination of surfaces in 30 different offices in three different cities (Tucson, New York, and San Francisco) found significant microbial diversity based on geographical location. The main sources of contamination were human and several of the bacterial genera found were pathogens (e.g., *Neisseria*, *Shigella*, *Streptococcus*, and *Staphylococcus*) [12]. These results suggest that potential pathogens may only be problematic for severely immune-compromised individuals in indoor offices. Furthermore, such studies shed significant scientific knowledge about the microbial diversity and possible health threats to humans living in sealed and enclosed environments for prolonged periods of time.

Humans have a reduced immune response when exposed to unfavorable environmental conditions like long-term confinement and spaceflight [13, 14]. In addition, several microorganisms have the capability to degrade habitat materials [9, 15, 16] and exhibited increased pathogenic characteristics during spaceflight [17]. Previous environmental microbiological investigations, like the one of the International Space Station (ISS), suggested that microbial species may undergo permanent changes, such as mutations in the genome over time, and consequently shift microbial population dynamics [5, 11, 18–20]. This finding emphasizes the importance of monitoring and preventing bio-contamination of enclosed environments inhabited by humans and measuring microbial succession to develop strategies in mitigating harmful microbial contamination [18]. The MARS 500 study’s microbiological estimation is the first full-duration simulation of a manned flight to Mars that measured microbiological changes of the air and surfaces using cultivation and molecular methods [21]. However, the MARS 500 study did not account for viable microorganisms utilizing iTag Illumina sequencing technology as reported here but rather adopted Sanger sequencing for evaluating both dead and live microorganisms. Moreover, the study did not include sampling of the facility prior to human occupation to determine a baseline and the change of the microbiome due to human presence.

In early 2009, a team led by the Department of Space Studies at the University of North Dakota (UND), USA, developed advanced inflatable habitat architecture concepts that could be adapted for use on surfaces of the Moon and Mars. An inflatable lunar/Mars analogous habitat (ILMAH) was built to occupy four student crews for mission durations up to 6 months. After completion of the habitat, three test subjects (graduate students of the UND; hereafter called student crews) occupied the ILMAH for two different periods of time (10 and 30 days) [22]. Among others, one purpose of the study was to identify psychological and behavioral problems related to a potential human mission to Moon or Mars. However, the study also provided the opportunity to measure microbiological changes during the student crew’s occupation of the ILMAH.

Here, we present the results of the microbial succession study in the ILMAH, which was conducted by monitoring the bacteriome of several surfaces before human occupation and during the 30-day occupation by the three student crew. To address the microbial divergence in closed indoor environments, surface samples from defined locations were sampled before and after the occupation, as well as three consecutive time periods during the habitation. In addition to the total bacteriome (iTag Illumina sequencing), cultivable and viable microbial populations of the ILMAH surfaces were elucidated. Viable but not-yet-cultivable microbial populations were determined by using well-established propidium monoazide (PMA) and adenosine triphosphate (ATP) assays [23, 24]. Microbiological characterization has been conducted for human habitats, such as indoor buildings [25, 26], hospitals [27], and the MARS 500 habitat [21]; however, this is the first study to characterize the viable microbial community of a closed habitat utilizing state-of-the-art technologies.

## Results

### Microbial burden

The microbial populations of various surfaces of the ILMAH, as estimated by culture-dependent and independent analyses, are summarized in Table 1. The cultivable bacterial counts ranged from 10^3^ to 10^7^ CFU/m^2^. After an initial decline of ~1 to 3 logs of colony counts from Day 0 (before occupation) to Day 13, the counts were exhibited an increase in order from Day 13, to Day 20, to Day 30 for locations in front of the sleeping area (bedroom) and bathroom, whereas the colony counts in the kitchen area decreased over these time points. However, the cultivable counts did not change during the 30-day occupation period in the lab locations. Changes in Day 0 cultivable bacterial population were significant (Additional file 1: Table S1), where Day 0 possessed a significantly higher cultivable population than the samples collected in subsequent time points (Table 1).

**Table 1:** Total, viable, and cultivable microbiological characteristics of ILMAH surface samples

Sample location	Cultivable bacterial population (CFU/m^2^)	QPCR-based bacterial population (16S rRNA copies/m^2^)			ATP-based microbial population (relative luminescence unit/m^2^)		
		(A) Total bacterial burden (PMA-untreated)	(B) Viable bacterial burden (PMA-treated)	Percentage of viable bacterial population (B/A × 100)	(C) Total microbial burden (total ATP content)	(D) Viable microbial burden (intracellular ATP content)	Percentage of viable microbial population (D/C × 100)
North Dakota ILMAH 30-day mission (before crew occupation)							
Bedroom 1	1.26 × 10^6^	1.44 × 10^6^	3.69 × 10^5^	25.68	3.93 × 10^6^	7.65 × 10^6^	>100
Bedroom 2	1.86 × 10^6^	1.10 × 10^6^	8.42 × 10^5^	76.62	1.27 × 10^6^	1.13 × 10^6^	88.89
Kitchen 3	4.63 × 10^6^	5.83 × 10^5^	6.23 × 10^5^	>100	9.80 × 10^5^	4.43 × 10^5^	45.16
Kitchen 4	4.25 × 10^6^	2.44 × 10^6^	1.50 × 10^6^	61.46	1.56 × 10^6^	2.28 × 10^6^	>100
Bathroom 5	1.41 × 10^6^	9.41 × 10^6^	1.70 × 10^6^	18.02	9.29 × 10^5^	3.36 × 10^5^	36.19
Laboratory 6	1.51 × 10^7^	1.33 × 10^6^	4.28 × 10^5^	32.23	2.15 × 10^6^	5.47 × 10^6^	>100
Laboratory 7	2.08 × 10^7^	6.43 × 10^3^	BDL	ND	2.03 × 10^6^	1.04 × 10^7^	>100
Laboratory 8	8.10 × 10^5^	BDL	BDL	ND	1.89 × 10^6^	1.02 × 10^6^	53.63
North Dakota ILMAH 30-day mission (Day 13)							
Bedroom 1	3.80 × 10^3^	1.23 × 10^6^	6.49 × 10^4^	5.28	7.82 × 10^5^	3.36 × 10^5^	43.04
Bedroom 2	1.85 × 10^4^	5.32 × 10^5^	7.80 × 10^4^	14.66	4.82 × 10^6^	6.63 × 10^5^	13.76
Kitchen 3	2.60 × 10^4^	8.88 × 10^5^	1.36 × 10^5^	15.28	6.96 × 10^6^	8.75 × 10^5^	12.58
Kitchen 4	7.35 × 10^4^	1.43 × 10^7^	4.66 × 10^5^	3.26	2.48 × 10^6^	1.20 × 10^6^	48.43
Bathroom 5	1.05 × 10^4^	7.95 × 10^5^	3.44 × 10^4^	4.33	3.50 × 10^6^	7.02 × 10^5^	20.07
Laboratory 6	8.93 × 10^4^	2.06 × 10^6^	6.46 × 10^5^	31.47	4.75 × 10^6^	1.89 × 10^6^	39.81
Laboratory 7	1.19 × 10^5^	4.16 × 10^6^	3.68 × 10^5^	8.85	4.60 × 10^6^	9.76 × 10^5^	21.22
Laboratory 8	3.33 × 10^4^	1.11 × 10^6^	3.25 × 10^5^	29.30	3.87 × 10^6^	1.03 × 10^6^	26.54
North Dakota ILMAH 30-day mission (Day 20)							
Bedroom 1	2.05 × 10^4^	6.93 × 10^5^	8.52 × 10^4^	12.31	6.04 × 10^6^	1.31 × 10^6^	21.65
Bedroom 2	1.62 × 10^5^	2.89 × 10^5^	4.45 × 10^4^	15.40	6.04 × 10^6^	1.78 × 10^6^	29.43
Kitchen 3	4.63 × 10^4^	4.38 × 10^6^	3.79 × 10^5^	8.66	5.05 × 10^6^	1.94 × 10^6^	38.48
Kitchen 4	2.10 × 10^4^	2.00 × 10^6^	1.01 × 10^5^	5.04	2.47 × 10^6^	5.97 × 10^5^	24.19
Bathroom 5	1.90 × 10^4^	2.84 × 10^5^	1.75 × 10^5^	61.86	4.05 × 10^6^	1.75 × 10^6^	43.15
Laboratory 6	5.58 × 10^4^	1.09 × 10^6^	2.16 × 10^5^	19.76	4.40 × 10^6^	2.10 × 10^6^	47.72
Laboratory 7	1.47 × 10^4^	3.71 × 10^5^	1.74 × 10^4^	4.70	2.65 × 10^6^	1.01 × 10^6^	38.05
Laboratory 8	5.55 × 10^4^	8.90 × 10^5^	1.86 × 10^5^	20.86	3.88 × 10^6^	1.47 × 10^6^	37.91
North Dakota ILMAH 30-day mission (Day 30)							
Bedroom 1	1.12 × 10^5^	3.81 × 10^6^	1.60 × 10^5^	4.19	1.61 × 10^6^	4.14 × 10^5^	25.77
Bedroom 2	2.33 × 10^5^	3.28 × 10^6^	3.38 × 10^5^	10.30	4.10 × 10^6^	1.83 × 10^6^	44.75
Kitchen 3	3.24 × 10^4^	3.98 × 10^6^	1.94 × 10^5^	4.88	2.42 × 10^6^	9.63 × 10^5^	39.85
Kitchen 4	2.15 × 10^3^	2.41 × 10^6^	8.69 × 10^4^	3.61	3.66 × 10^6^	3.61 × 10^5^	9.87
Bathroom 5	1.03 × 10^5^	3.29 × 10^6^	1.44 × 10^5^	4.38	2.17 × 10^6^	6.15 × 10^5^	28.37
Laboratory 6	1.27 × 10^5^	5.92 × 10^6^	1.09 × 10^6^	18.36	1.48 × 10^6^	1.07 × 10^6^	72.35
Laboratory 7	6.30 × 10^4^	6.89 × 10^6^	7.78 × 10^5^	11.30	2.96 × 10^6^	1.25 × 10^6^	42.19
Laboratory 8	5.85 × 10^4^	4.86 × 10^6^	3.41 × 10^5^	7.02	4.12 × 10^6^	7.29 × 10^5^	17.68

*BDL* below detection limit, *ND* since one of the values is BDL, the ratio cannot be determined

The qPCR assay that measured 16S ribosomal RNA (rRNA) gene copies from both dead and live bacterial cells showed an increase in bacterial density over the 30-day occupation period. In general, samples treated with PMA (viable bacterial burden) revealed that ~60 % of the bacterial population was dead at Day 0, whereas the reduction in bacterial population was ~90 % for the subsequent days of occupation. The viable bacterial burden as measured by the PMA-qPCR assay ranged from below detection limit to 10^6^ 16S rRNA gene copies/m^2^. Furthermore, during the 30-day occupation period, the ratio of cultivable bacteria to viable bacterial burden (ATP assay results) was at least twice more on the final day (Day 30; 33.4 %) than before occupation (Day 0; 18.9 %). The percent cultivable bacteria among viable bacterial burden were greater at the surfaces in front of the bathroom (~47 %) or in bedroom area (~55 %) when compared to other locations (~12 to 14 %). These differences were highly significant when considering qPCR results of PMA vs. non-PMA samples in a paired *t* test (*p* = 0.000106).

The total (ATP content from both dead and live microbes) and viable microorganisms (intracellular ATP content) were in the range of 10^5^–10^6^ relative luminescence unit per m^2^. In general, when samples from all sampling locations were pooled together, the total microbial burden did not show any changes in microbial accumulation over the time period. However, ~55 % of the microbes were viable at Day 1, and the viable microorganisms were reduced to ~28 % on Day 13 and ~35 % on the last day of occupation. The percentage of viable bacterial burden (PMA-qPCR assay) among viable microbial burden (intracellular ATP content) was highest in the lab area (~40 %) and lowest in the bathroom area (~10 %). A closer look at the microbial burden as measured by ATP content of the different locations revealed that the succession of bacterial burden was not linear throughout the 30-day occupation. The bacterial density initially decreased in all the locations (from Day 13 to Day 20) and then drastically increased (Day 30) in the bedroom area, the bathroom area, and the lab area, whereas samples from the kitchen area showed no fluctuation. From a statistical standpoint, the microbial density fluctuations in various locations were not significant (Additional file 1: Table S2).

### Cultivable bacterial diversity

**Fig. 1:**
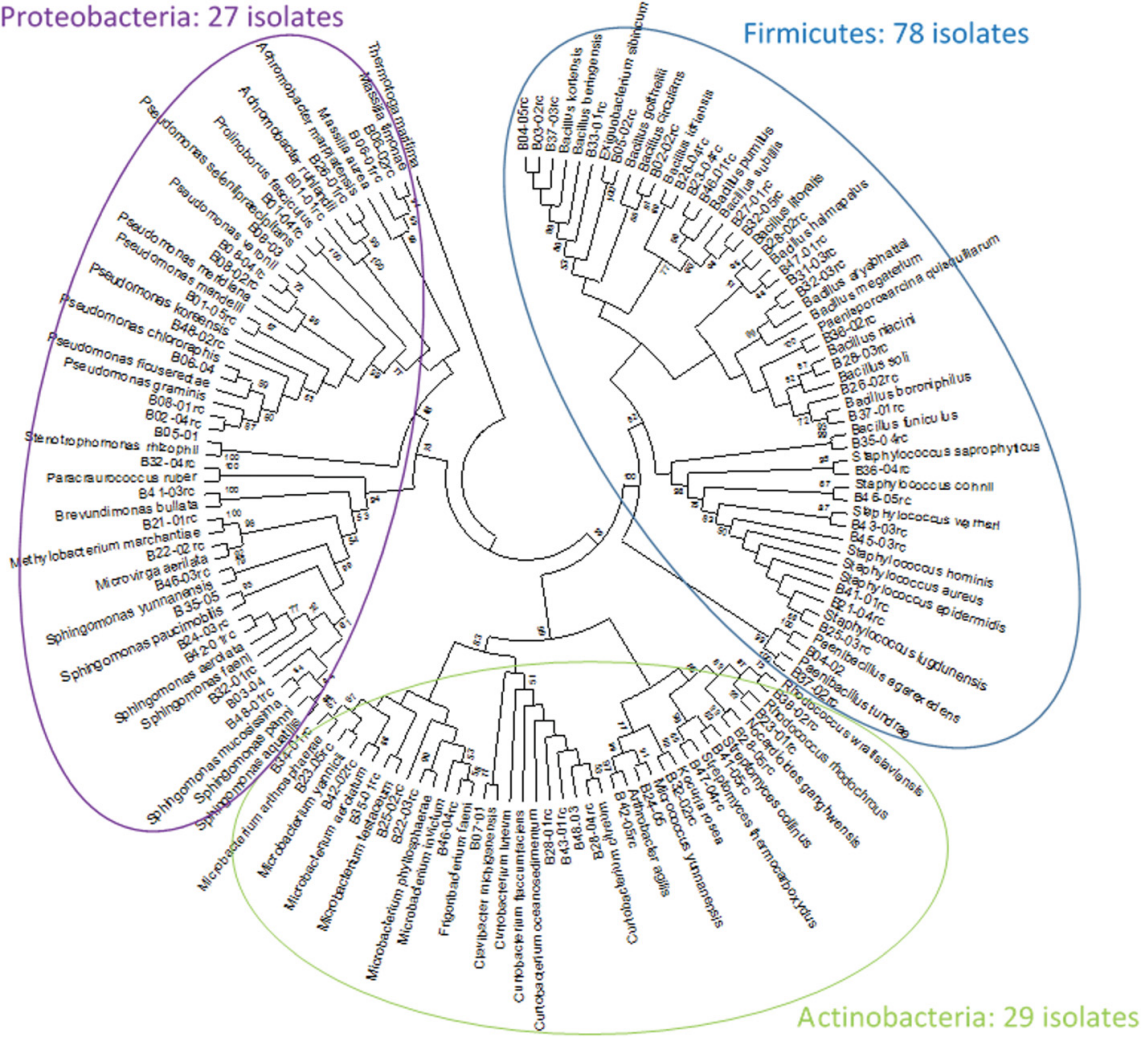
Cultivable bacterial diversity detected through the 30-day habitation period at all the locations based on 16S rRNA gene sequences. The strain designation or type strain of the closest neighbor followed by the GenBank EZtaxon-e is given. The phylogenetic tree was constructed using neighbor-joining method (bootstrap 1000). In total, 150 isolates were collected, 147 of which were successfully sequenced (3 strains did not respond to the sequencing methods attempted and hence excluded from the tree). The numbering of the isolates is explained as follows. *B* = bacteria, first number (0–4) will be the sample collection day (0 = Day 0, 2 = Day 13, 3 = Day 20, 4 = Day 30), second number (1–8) will be sampling location and the third number (01–05) is the replicate number of the isolate. For example, B-38-02 will be a bacterial strain, isolated from Day 30, at location number 8, and a second isolate. Frequency of isolates is given in *parenthesis* after the name of the bacterium

Phylogenetic characterization of 150 strains isolated during this study and identified via 16S rRNA gene analysis revealed a total of 62 known bacterial species and eight lineages yet to be described (based on 97 % similarity of 16S rRNA genes to publicly available sequences; Fig. 1, Additional file 1: Table S3). More than half of the identified isolates belonged to *Firmicutes* (76 strains), 26 % to *Proteobacteria* (39 strains), and 20 % to *Actinobacteria* (30 strains). *Bacillus* species represented the highest number of isolates (43 strains), followed by *Staphylococcus* (24 strains) and *Pseudomonas* (17) species.

Throughout the occupation period, changes in the abundance of the cultivable bacterial species occurred. Before the student crew moved in (Day 0 sampling), almost two thirds of the isolates were *Proteobacteria* (14 strains), 28 % *Firmicutes* (8 strains), 8 % *Actinobacteria* (2 strains), and 3 % *Bacteroidetes* (1 strain). The majority of the proteobacterial isolates (13 out of 14 strains) belonged to *Gammaproteobacteria* and most *Firmicute* isolates (6 out of 8 strains) were *Bacillus* species.

At Day 13, the relative abundance pattern of cultivable bacteria changed drastically. The frequency of *Proteobacteria* declined to 10 %, whereas *Firmicutes* and *Actinobacteria* increased to 60 and 29 %, respectively. None of the *Gammaproteobacteria* recovered at Day 0 were retrieved at Day 13 (or Day 20 or Day 30), instead *Alphaproteobacteria* was present. A few of the *Bacillus* species found at Day 0 recurred at Day 13 (*Bacillus idriensis*, *Bacillus litoralis*, and *Bacillus niacini*) and additional *Bacillus* species were isolated (e.g., *Bacillus soli*, *Bacillus megaterium*). *Actinobacteria* that were recovered for the first time were represented by the genera *Microbacterium*, *Curtobacterium*, *Micrococcus*, and *Rhodococcus*.

Day 20 samples showed a similar relative abundance pattern of the phyla as the Day 13 samples. No species of *Proteobacteria* and almost no species of *Actinobacteria* (one exception: *Curtobacterium faccumfaciens*) from Day 13 were isolated again at Day 20. However, bacterial species from all of the same genera at Day 13 also recurred at Day 20. Some of the *Firmicutes* from Day 13 reappeared, whereas some *Bacillus* species emerged for the first time.

Day 30 samples revealed a slight change in the relative abundance pattern compared to Day 20: less *Firmicutes* (51 %) were retrieved in favor of *Proteobacteria* (21 %). The recovery rate of *Actinobacteria* did not change (24 %), and one *Bacteroidetes* was isolated (3 %). Consistently, almost no *Proteobacteria* and *Actinobacteria* from the earlier time points were detected again. However, other species of the previously recovered genera were found and some of the *Firmicutes* from Day 13 and/or Day 20 were isolated again and some undetected *Firmicutes* species emerged (Fig. 1).

The analysis of the cultivable bacteria indicates a strong decline in the number of *Proteobacteria* from Day 0 to Day 13 and an increase in *Firmicutes* and *Actinobacteria* over this time. Changes within the later three time points (Day 13, Day 20, and Day 30) were only detectable at species level, not at phyla level. Almost all representative species from *Actinobacteria* and *Proteobacteria* were isolated only once throughout the whole occupation period, whereas representatives of the *Firmicutes* were recovered multiple times.

### Controls

Sampling device control, environmental controls, DNA extraction reagent controls, and no-template PCR controls included in this study yielded no sequence reads. The colony counts, qPCR, and ATP-based analyses to estimate microbial burden also exhibited values below detection limit.

### Significant differences in viable and total bacteriome

**Fig. 2:**
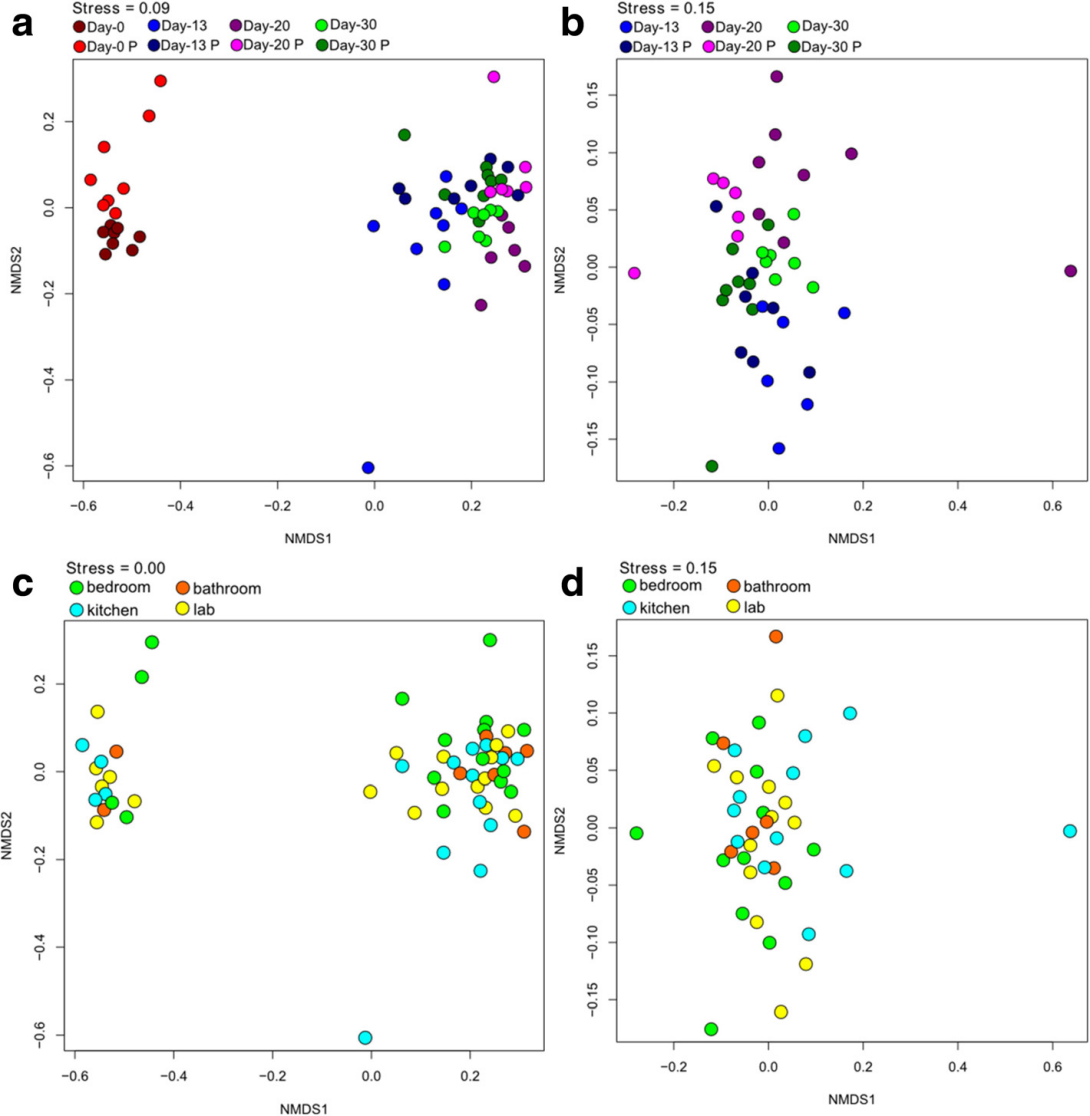
NMDS ordinations based on Bray-Curtis distances between all samples; **a** NMDS ordination displaying the distance between samples taken at the different time points; **b** NMDS ordination displaying the distance between samples taken at the different time points excluding D0 samples; **c** NMDS ordination displaying the distance between samples taken at the different locations; Adonis *p*-value 0.452, MRPP, significance of delta 0.957, A: –0.02321. **d** NMDS ordination displaying the distance between samples taken at the different locations excluding D0 samples; Adonis *p*-value 0.486, MRPP, significance of delta 0.254, A: 0.005762. A suffix “P” after the respective time points indicates that these samples are treated with PMA. **a**, **b** Various time points: Adonis *p* = 0.001 and MRPP significance of delta = 0.001 and A = 0.02813. **c**, **d** Various locations: Adonis *p* value of 0.452, MRPP, significance of delta = 0.957, A = −0.02321. The permanova and MRPP when looking at two different test (being treated with PMA or being not treated with PMA) are Adonis *p* = 0.034 and MRPP, significance of delta = 0.023, A = 0.01888. The Day 30; Kitchen-4 (see Table 1) sample is a potential outlier but removing this sample from the NMDS permutations did not change the significance values of the different variables but this sample was not included in this figure

The bacterial richness between PMA and non-PMA samples declined significantly (paired *t* test based on number of operational taxonomic units (OTUs) revealed a *p* value <0.0001). Moreover, 208 genera were detected in the non-treated samples, 37 of which were not identified in the PMA-treated samples. The two sample types also differed significantly in community relationships (NMDS analysis in Fig. 2a, c, Adonis *p* = 0.034 and MRPP, significance of delta = 0.023, A = 0.01888) and their Shannon diversity index indicated a significant reduction (paired *t* test *p* < 0.001). Due to these differences in the total and viable bacteriome, it could be concluded that the total bacteriome (including dead cells) does not give a true picture of the causative bacterial agent(s) that trigger illness. Consequently, all following results are based on data generated from the viable bacteriome only.

### Substantial changes in the viable bacterial community composition after occupation

The diversity of the overall viable bacteriome was dominated by *Actinobacteria*, *Firmicutes*, and *Proteobacteria* (97 % of all characterized OTUs) and, in combination with the *Bacteroidetes*, these four phyla accounted for 99 % of the characterized OTUs. A closer examination of the OTUs on genus level indicated a predominance of nine taxa, which were primarily comprised of by *Corynebacteria* (20 % of total OTUs), *Bacilli* (15 %), *Staphylococci* (18 %), *Anaerococci* (11 %), and *Fusobacterii* (14 %). In sum, the sequences from the aforementioned five genera constituted 78 % of all OTUs found in the PMA-treated samples (Table 2).

**Table 2:** Bacterial taxa retrieved from ILMAH surfaces sampled at various time points of human occupation

Bacterial taxon	Number of sequences from sampling time at							
	Day 0		Day 13		Day 20		Day 30	
	Total	Viable	Total	Viable	Total	Viable	Total	Viable
Acidobacteria	15	11	89	47	15		49	41
Actinobacteria	2803	1156	17631	10697	14865	4806	23314	17663
Bacteroidetes	2733	538	2418	169	1719		1517	538
Candidatus Saccharibacteria	91	52	106	39	56		99	7
Chlamydiae	22	23	31				8	
Chloroflexi	94	42	49	59	19	28	43	11
Cyanobacteria	129	53	567	29	96		1704	541
Deinococcus-Thermus	50	9	7	1	3	2	4	
Firmicutes	6303	4719	17282	9604	13910	2212	23312	15422
Fusobacteria	11		54	1	13		111	5
α-proteobacteria	15956	4082	2959	1196	396	29	1893	1881
β-proteobacteria	10664	3051	1835	980	261	39	1242	1065
δ-proteobacteria	21	5	113	88	34	8	13	16
ε-proteobacteria	3		6	1			2	2
γ-proteobacteria	3088	1279	3561	1777	3997	27	2340	412
Parcubacteria			19	54	3		5	13
Others (4 phyla)	1	3	21	20	5		19	3
Unclassified bacteria	131	63	247	620	71	10	215	150

To investigate differences in the bacteriome between samples, multivariate statistics were applied using ordination analyses and Monte Carlo-based permutation tests. Viable bacterial communities formed significantly different groups in NMDS plots based on the factor time, i.e., the different sampling days showed distinct microbiome profiles (Fig. 2a, various time points: Adonis *p* = 0.001 and MRPP, significance of delta = 0.001 and A = 0.02813; Fig. 2c, various locations: Adonis *p* value of 0.452, MRPP, significance of delta = 0.957, A = −0.02321). Notably, the bacteriome of Day 0 samples was substantially different from the bacteriome of the later time points. For that reason, Day 0 samples were excluded in another analysis (Fig. 2b, d), which also revealed that the community profiles of the later three sampling events (Day 13, Day 20, and Day 30) were significantly different from each other, even though the differences were smaller compared to Day 0 (Fig. 2b; various time points: Adonis *p* = 0.001 and MRPP, significance of delta = 0.001 and A = 0.06871). The NMDS ordinations based on Bray-Curtis distances between all samples that were not treated with PMA (Additional file 2: Figure S2) and PMA (Additional file 2: Figure S3) are shown. The UniFrac distance of various ILMAH bacteriome datasets are shown in Additional file 2: Figure S4. The bacteriome distribution patterns were similar whether Bray-Curtis distance or UniFrac distance was used (UniFrac for various time points: Adonis *p* value of 0.001, MRPP significance of delta = 0.001, A = 0.2988; UniFrac for various locations: Adonis *p* value of 0.801, MRPP significance of delta = 0.955, A = −0.03882; different treatments (PMA and no PMA): Adonis *p* value of 0.012, MRPP significance of delta = 0.004, A = 0.03886).

Due to the differences in multivariate statistics, the bacteriome change on a single OTU level was investigated. Prior to the occupation (Day 0 sampling), the majority of the microbial community consisted of *Firmicutes* (59 %), followed by *Proteobacteria* (27 %), and *Actinobacteria* (9 %). By the end of the occupation (Day 30 sampling), the relative abundance of *Firmicutes* in the habitat had not changed significantly (61 %), whereas *Proteobacteria* decreased (16 %) and *Actinobacteria* increased (21 %) (Table 2). The bacteriome profiles of various locations of ILMAH surfaces are shown in Table 3.

**Table 3:** Bacterial taxa retrieved from ILMAH surfaces sampled at various locations during the human occupation

Bacterial taxon	Number of sequences from							
	Bedroom		Kitchen		Bathroom		Lab	
	Total	Viable	Total	Viable	Total	Viable	Total	Viable
Acidobacteria	83	2	43	44	16	47	26	6
Actinobacteria	22177	5326	20602	14580	6168	3764	9666	10652
Bacteroidetes	2511	73	3733	396	613	55	1530	721
Candidatus Saccharibacteria	147		78	50	81	10	46	38
Chlamydiae	35		9		10	14	7	9
Chloroflexi	57	6	86	91	16	8	46	35
Cyanobacteria	396	4	1845	37	107	13	148	569
Deinococcus-Thermus	5		39	7	2	1	18	4
Firmicutes	22694	5259	21324	13813	6557	3323	10232	9562
Fusobacteria	43	1	124		11		11	5
α-proteobacteria	6737	825	8090	2586	1384	712	4993	3065
β-proteobacteria	4321	1062	5303	2236	760	363	3618	1474
δ-proteobacteria	71	9	46	100	51		13	8
ε-proteobacteria	5		5		1	1		2
γ-proteobacteria	1661	292	8988	2121	560	200	1777	882
Parcubacteria	19		5	54		13	3	
Others (4 phyla)	4		24	23			18	3
Unclassified bacteria	180	58	301	669	42	33	141	83

**Fig. 3:**
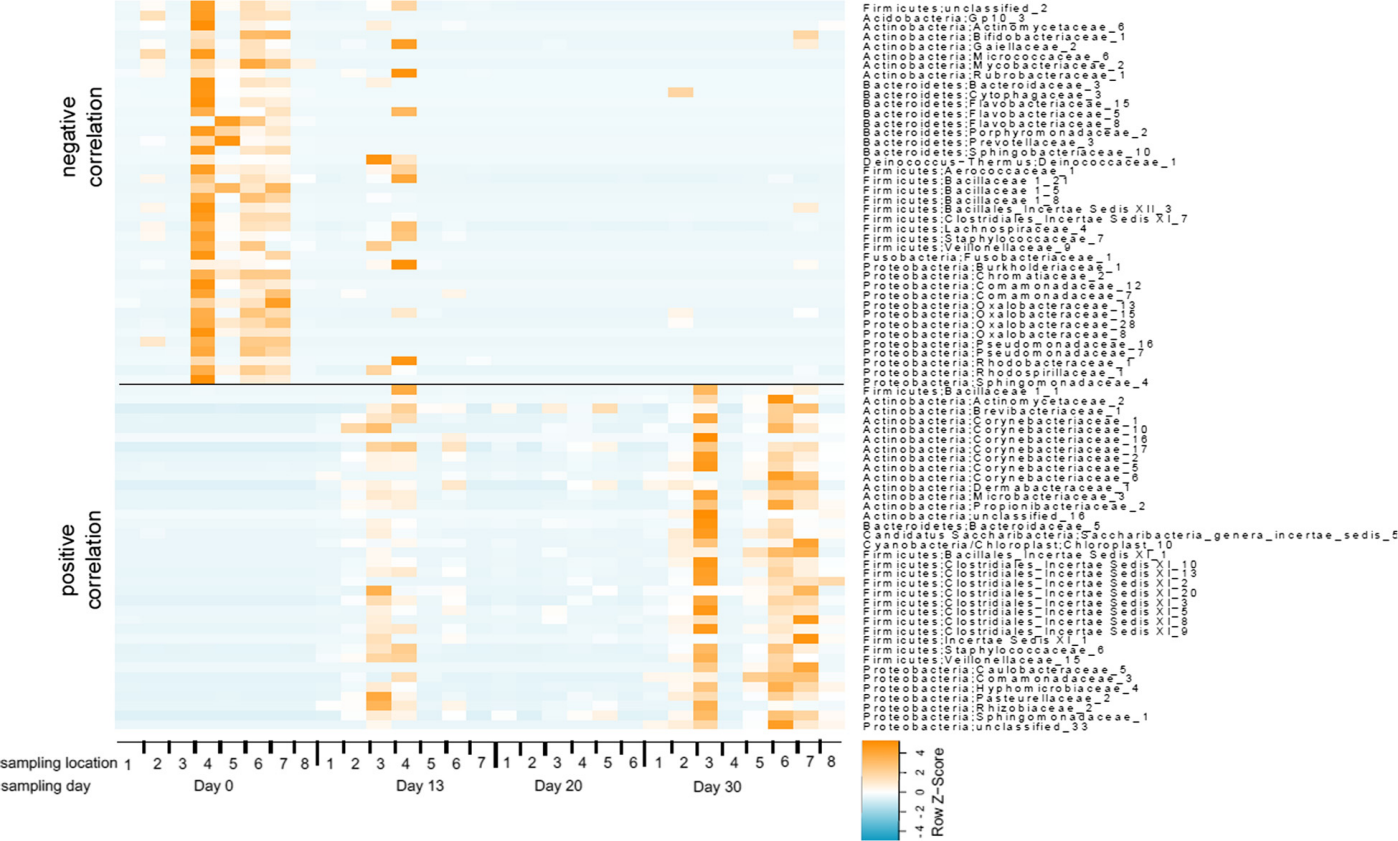
Heat map of the taxa that showed a significant correlation (*p* value of 0.01) with the factor time in the PMA-treated sample set. The color *blue* indicates a low abundance of the single OTU in the respective sample and *orange* indicates a high abundance of the single OTU in the respective sample

To identify viable taxa that significantly correlated with the various occupation time points, a Spearman rank correlation was applied individually to each OTU’s abundance pattern and sampling time. The results are displayed as a heat map (Fig. 3, PMA-treated samples) presenting only those OTUs that showed a significant correlation (76 in number) with a *p* value of 0.01. Most of the OTUs belonged to the phylum *Firmicutes* (23 OTUs), followed by *Proteobacteria* (20 OTUs) and *Actinobacteria* (19 OTUs); the rest of the taxa were classified as *Bacteroidetes* (9 OTUs) and *Candidatus Sachcharibacteria* (formerly known as TM7), *Cyanobacteria*, *Deinococcus*-*Thermus*, *Fusobacteria* and *Acidobacteria* (1 OTU, respectively). *Actinobacteria* and *Firmicutes* that increased throughout the occupation period were dominated by two families: *Corynebacteriaceae* (7 OTUs), *Clostridiales Incertae Sedis XI* (8 OTUs). Other OTUs that increased throughout the occupation period were mostly *Proteobacteria* (7 OTUs) belonging to different families. Members of *Proteobacteria* that decreased throughout time (13 OTUs) were dominated by *Oxalobacteraceae* (4 OTUs), *Comamonadaceae* (2 OTUs), and Pseudomonadaceae (2 OTUs). Among *Firmicutes* (9 OTUs), members of the family *Bacillaceae* (3 OTUs) increased and all but one *Bacteroidetes* (8 OTUs) decreased within the course of time.

Since the Day 0 samples exhibited a fundamentally different bacteriome composition, two additional analyses were employed. First, Spearman rank correlation was applied on the PMA-treated dataset excluding Day 0 samples. At a *p* value of 0.05, 26 OTUs showed a significant correlation with the factor time, six of which decreased throughout the occupation period, whereas 20 OTUs increased. Most of the OTUs that exhibited a positive correlation belonged to the phyla *Proteobacteria* (8 OTUs; dominated by Caulobacteraceae (2 OTUs)), followed by *Firmicutes* (7 OTUs; dominated by *Clostridiales Incertae Sedis XI* (4 OTUs)), followed by *Actinobacteria* (3 OTUs), and *Actinobacteria* (2 OTUs). Of those 26 OTUs, three families were identical with the correlation analyses above, confirming that OTUs belonging to *Clostridiales Incertae Sedis XI*, *Comamonadaceae*, and *Corynebacteriaceae* truly increased.

**Fig. 4:**
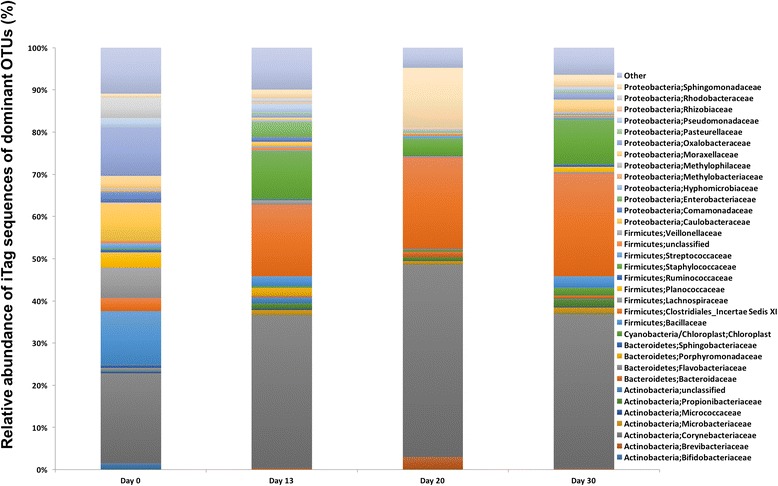
Dominant microbial population and microbial succession patterns observed in 30-day occupation period of the ILMAH system. The OTU abundance that are >0.75 % of the total microbial population are computed in this bar graph. Day 0 surfaces show completely different microbial profile when compared to the subsequent sampling days of the crew occupation

Second, an ANOVA was applied on OTU abundances across all the PMA-treated samples comparing data at Day 0 with those at the later three time points (*p* = 0.01). Thirty-four OTUs that were more abundant at Day 0 were identified as belonging to similar genera as OTUs identified in the correlation analysis. They belonged mainly to *Oxalobacteraceae* (8 genera) and different genera belonging to *Proteobacteria*, *Actinobacteria,* and *Firmicutes*. The predominant and differential bacterial taxa (>0.75 % of overall microbial abundance) of various time points of the ILMAH surfaces are depicted in Fig. 4.

In sum, statistical analyses revealed a significant difference in community structure of samples over time, particularly of the bacteriomes existing before human occupation of the habitat (Day 0 sampling) and after occupation (Day 13, Day 20, and Day 30 samplings). *Actinobacteria* (mainly *Corynebacteriaceae*) and *Firmicutes* (mainly *Clostridiales Incertae Sedis XI* a) were shown to increase over the occupation time period.

### Archaea

In general, archaea were of low abundance in the samples collected from the ILMAH surfaces. The archaeal microbial burden detected via specific qPCR was below detection level in most of the samples tested (28 out of 32 samples) and in positive samples, archaea were between one and two logs lower than the bacterial microbial burden. The overall low abundance of archaea in the community was confirmed by the iTag data, since 92 % of all the OTUs found with archaea-specific primers were identified as bacteria indicating low abundance of archaea resulting in unspecific priming. Due to the low number of sequences detected it was not possible to depict any change in the archaeal community structure throughout the occupation period (Additional file 1: Table S4). It is important to note that *Nitrososphaera* represented the majority of all OTUs found in the non-PMA-treated samples (94 %). Along with *Nitrososphaera*, three other archaeal genera were detected: *Methanocaldococcus*, *Methanosarcina*, and *Nitrosopumilus.* However, in the PMA-treated sample set, *Nitrososphaera* was the only archaeal genera observed, indicating that the latter three genera might be dead highlighting the importance of using viability assays for archaea in indoor microbiomes.

## Discussion

Human missions to other celestial bodies like the Moon or Mars are currently being planned for the future by space-faring nations [28]. When building a self-sufficient settlement, microbial accumulation of viable cells that may potentially cause disease will be a concern for crewmembers’ health. Application of validated microbial reduction technologies for a closed habitat may substantially reduce microbial populations [29] but leave behind genetic materials that could be falsely diagnosed for the presence of potentially dangerous biological contamination. Hence, it is necessary to develop microbial detection technologies to target viable cells that cause disease (human and plants) and deteriorate the human habitat. Findings from microbiological characterization of the controlled Mars analogous habitat (ILMAH, MARS 500, etc.) and other closed systems (ISS) will help space agencies in developing appropriate countermeasures to eradicate viable microorganisms that could be problematic to human health.

Previous studies have shown that meaningful data can often only be retrieved if the dead fraction of the cells is excluded [23, 30]. Otherwise, the significance of viable, but low in abundance microbial communities might be underestimated, because their molecular signals are masked by other taxa that are dominant but dead. As shown in this ILMAH study and, other reports, more than 50 % of the cells were dead and the bacterial diversity was reduced significantly in samples treated with PMA [11, 23]. In addition, low-abundant bacterial taxa were more present in the PMA-treated samples when compared with the non-treated samples, which is statistically significant (*p* = 0.04813) and had been reported before for a variety of microorganisms including viruses [31]. This approach confirmed that a PMA treatment or other validated life/dead-detection method is essential when characterizing molecular microbial communities, thus eliminating the nucleic acids from dead cells. In this study, only the viable microbial community structure was discussed in terms of predicting changes in microorganisms over the successive sampling time points.

Various studies report that human presence is the most common source of contamination in strictly controlled rooms [21, 32, 33]. Dispersal of microorganisms by humans is dependent on their activities and time spent in the closed habitats. Typically, human natural skin renewal and shedding are 10^6^ to 10^7^ particles per day [34]; perspiration, coughing, or speaking expels 10^3^ to 10^4^ droplets per (re)action; [35, 36]. In addition, shoes and clothing introduced into a controlled room by the inhabitants are the other sources of microorganisms [37]. The ILMAH study results also indicate that the presence of humans influenced the microbial diversity and composition in the closed habitat. Most of the observed variations in the ILMAH microbial community were due to the move-in of the student crew. Both the cultivable bacteria and the analysis of the bacterial community via iTaq sequencing showed that samples taken at Day-0 (before occupation) contained an ecologically distinctive set of microbial taxa that were not abundant in the samples taken at Day-13, -20, and -30 (Fig. 4). Microbial community changes within the later three time points (Day-13, -20, and -30) were seen only at species level, not at phyla level. It is becoming increasingly evident that quorum sensing enhances the ability of bacteria to access more favorable environmental niches and increase bacterial defenses against eukaryotic hosts, competing bacteria, and environmental stresses [38]. The physiological and clinical aspects of quorum sensing have received considerable attention and are starting to be studied at the molecular level. However, little is known about whether quorum sensing plays an important role in indoor environments. Additional research is needed to understand the mechanism(s) of biofilm formation by the predominant environmental microbial species of the closed systems and the influence of cell-to-cell signaling. With a greater understanding, it may be possible to maintain an environment that interferes with quorum sensing, thus inhibiting growth of potential corrosive microorganisms, virulence, and biofilm formation, which would greatly benefit the health and safety of humans in closed systems [39].

The viable bacterial communities (PMA-treated samples) retrieved from samples at Day 0 (*Flavobacteriaceae*, *Caulobacteraceae*, and *Oxalobacteraceae*) were reported to be common in aquatic and soil habitats [40, 41]. The 10 % bleach cleaning solution used on the ILMAH surfaces was likely detrimental to these bacteria and eradicated most of the soil microorganisms documented in Day 0, since their presence in subsequent sampling periods was not in great abundance (Fig. 4). The bacterial communities noticed at Day-13, -20, and -30 were dominated by taxa mostly associated with humans as commensals or pathogens, such as *Corynebacteriaceae*, *Clostridiales Incertae Sedis*, and *Staphylococcaceae* [42–45], and were reported to be associated with the human skin or gut microbiome [46]. *Actinobacteria* (*Corynebacterium, Propionibacterium*, etc.) were more abundant on skin, with *Firmicutes* (*Clostridium*) and *Bacteroidetes* more abundant in the gastrointestinal tract. Sequences of notable pathogens, such as *Brevibacteria* (osteomyelitis and otitis; [47]), *Actinomycetae* (actinomycosis; [48]), *Propionibacteria* (acne; [49]), *Corynebacteraceae* (nosocomical; [50–52]), *Staphylococcaceae* (skin rashes; [53]), and *Clostridium* (tetanus and botulism; [54]) were retrieved from the samples that were treated with PMA and thus may be viable. Even though sequences of potential pathogens were retrieved from the ILMAH surfaces and phylogenetically identified, their pathogenicity could not be confirmed with the available data. Future metagenomic study might shed light on the presence of the virulence genes in addition to the phylogenetic signatures.

Several studies have already been conducted on the microbial population in confined habitats, including several office buildings [12, 55, 56], hospitals [57], the Concordia Research Station [9], spacecraft surfaces and associated cleanrooms [7, 58–60], the MARS 500 habitat [21], and the ISS [5, 11, 19]. These studies also confirm that the sources of microbial contamination are human and opportunistic pathogens able to thrive in enclosed environments. All monitored confined habitats reported having restricted waste disposal and limited fresh air supply, which may possibly lead to bad air quality, water condensation, and accumulation of biological residues [9, 21]. Unlike human-inhabited enclosed environments, cleanrooms (pharmaceutical, medical, and spacecraft assembly) restrict human access (e.g., generally, ~8 h per shift). Various researchers have investigated these highly maintained cleanroom surfaces using iTag sequencing and metagenomic analyses [31, 59, 60] and found that the majority of the microbial contaminations were of human origin despite restricted human access. However, when specific cultivation-based assays were performed, domination of spore-forming bacteria was reported in these cleanrooms [4, 61–64]. The cultivable bacterial burden was significantly higher in ILMAH surfaces before occupation (mean 4.9 × 10^6^/m^2^) compared to the occupation period (Day 13, mean 3.8 × 10^4^/m^2^). The bacterial burden in cleanrooms was comparatively low (1- to 3-logs less) than the ILMAH and MARS 500 habitats [21] and may be attributed to the relatively low human activity, enforced dress regulations, and controlled entrances.

Of all the microbial characterizations studies in confined habitats, the MARS 500 project (June 2010 to November 2011) had the most similar study objectives to the ILMAH study. However, the MARS 500 study did not measure the viable portion of the community. Hence, the measured bioburden and diversity characterization of the MARS 500 report is an overestimation. This has been confirmed since the MARS 500 study revealed a pattern: members of the class *Proteobacteria*, followed by *Bacteroidetes* and *Firmicutes*, decreased over the occupation period; this was also noticed in the ILMAH samples that were not treated with PMA (including both dead and viable bacteria). But such trend was not found for the viable community of the ILMAH. Furthermore, the MARS 500 microbial analysis did not contain control, i.e., preoccupation bacterial diversity measurements, to compare the microbial accumulation as measured in this study. We conclude that it is critical to compare the microbiome of habitat without human occupation to habitat with human occupation since humans appeared to be the primary source of contamination in such habitats. Such a comparison may enable development of cleaning and maintenance protocols. Nonetheless, the MARS 500 project [21] provided important insight about the succession of a microbial community over time and, when compared to this study, the need for live/dead differentiation protocol(s) to elucidate the presence of viable pathogens in closed systems for prolonged periods of time become essential. These studies would help space-faring nations mitigate microbiological problems by developing countermeasures to eradicate unwanted microbial pathogens.

However, the microbial risk assessment in an Earth setting might underestimate the importance of humans living in stressed situations, such as microgravity and long-term travel in confined spaces [65]. Under microgravity conditions, the human immune system is compromised [13, 14] and bacteria exhibit enhanced virulence, antibiotic resistance [17, 66], and increased biofilm formation [67, 68]. The microbial monitoring of the ISS by various space-faring agencies revealed that *Staphylococcus* and *Aspergillus* were the dominant cultivable species [5, 69]. However, state-of-the-art molecular techniques to elucidate viable microbial communities of various ISS surfaces and microbial accumulation and succession (biofilm formation) analyses are warranted. The recent ISS air filters and vacuum debris analyses showed that cultivable *Staphylococcus* species are dominant but also reported retrieval of a large percent (>90 %) of molecular signatures of viable *Corynebacterium/Propionibacterium* [11].

Studies on archaea have concentrated on natural biotopes where they have been found in overwhelming numbers and with versatile properties [70]. Moreover, extremophilic archaea are considered the terrestrial life most likely capable of surviving on Mars [71], thus it is important to characterize the archaeal community in closed habitats such as the ILMAH. The role of archaea in artificial, human-controlled environments is still unclear, since there are few studies monitoring archaea in households, offices, airplanes, clinical environments, or other restricted environments like pharmaceutical and industrial cleanrooms and spacecraft assembly cleanrooms [72–74]. Previous archaeal surveys suggest that spacecraft assembly cleanroom facilities inhabit a restricted diversity of archaea. Examination of cleanrooms in Europe, South America, and the USA found archaeal signatures belonging to *Thaumarchaeota* and *Euryarchaeota* in one third of the collected surface samples (and none from the air samples). In this ILMAH study, only 4 out of the 80 samples (5 %) tested positive for archaea. The majority of the ILMAH archaeal community comprised OTUs of *Thaumarchaeota* (over 94 %) and the rest were *Euryarchaeota* (6 %), which supports the results from the aforementioned studies. Particularly, *Thaumarchaeota* have been recognized to inhabit human skin, another indication that the ILMAH microbiome was influenced by human activity [74].

## Conclusions

Accumulation of cultivable and viable (as per molecular methods) bacteria was evident during the 30-day occupation period of the enclosed habitat. No observed change in the total microbial burden (including dead cells) stressed the importance of differentiating dead organic matter from live cells. Thorough maintenance procedures adapted to keep the ILMAH system clean might have eradicated the microorganisms but such processes would not have removed the biomolecules from the surfaces, hence implementation of genetic methods estimating total microbial community structures including dead cells would result in overestimation. However, when viability assays were used, only ~55 % of the microbes were viable at Day 0, and they were reduced to ~28 % on Day 13 and ~35 % on the last day and this might be due to the prolonged stay by the human and their microbial shedding. Among the cultivable bacterial diversity members of the *Firmicutes*, *Proteobacteria*, and *Actinobacteria* phyla were dominant and such results also supported by the Illumina-based sequencing studies. Changes in the abundance of the cultivable bacteria and molecular signatures of viable bacterial species were noticed throughout the occupation period and are statistically significant. *Actinobacteria* (mainly *Corynebacteriaceae*) and *Firmicutes* (mainly *Clostridiales Incertae Sedis XI* and *Staphylococcaceae*) were shown to increase over the occupation time period. As seen in other studies where confined habitats were investigated, this study also concluded that humans are the primary source of contamination. A combination of cultivation-based analysis and viability assays is warranted to elucidate the significance of bioaccumulation that might be problematic to the inhabiting human health.

## Methods

### Sample locations and sampling

#### Habitat

The ILMAH is approximately 12 m long, 10 m wide, and 2.5 m high. The ILMAH interior consists of four sleeping compartments where the student crews are able to rest as well as stow their personal belongings, a small galley/dining room, a bathroom, and plenty of laboratory space (Additional file 2, Figure S1). Three student crews inhabited the ILMAH for 30 days collecting several surface samples at four different time points for further microbiological analyses.

#### Habitat preparation and cleaning procedures

Twenty-four hours prior to inhabitation, the interior surfaces of the habitat were cleaned with a 10 % bleach solution. The cleaning reagent used was a prediluted, stabilized sodium hypochlorite solution to disinfect hard surfaces in labs and production areas. The product is made up of 0.525 % sodium hypochlorite (1:10 bleach solution), the strength recommended by the Center for Disease Control for inactivating viruses and other pathogens. Immediately after cleaning, the ILMAH habitat was closed and undisturbed until 10 min prior to inhabitation. The student crews cleaned all the rooms and surfaces of the habitat once a week after they had taken the microbial samples. Cleaning included wiping down the surfaces (kitchen, tables, and bathroom) with antibacterial wipes (Catalog #:TX3214; Texwipes, Kernersville, NC), dusting, sweeping, and wet mopping the floor. Finally, the student crews performed additional cleaning duties, as required to keep the ILMAH system tidy. Cleaning procedures utilizing bleach, as carried out prior to occupation (Day 0), were not allowed during human occupation.

### Sample location, collection, and processing

The architectural scheme of the ILMAH system is depicted in Additional file 2: Figure S1. All samples were collected from the ILMAH floor surfaces. Among the eight prescribed locations (1 m^2^ each), two sample locations were in the sleeping compartment area, two in the dining room area, one in front of the bathroom, and three in the lab area. Samples from the closed habitat were collected using premoistened biological sampling kits (BiSKits; QuickSilver Analytics, Abingdon, MD, USA) from the eight prescribed locations at four time points (Day 0, Day 13, Day 20, and Day 30). Sampling point Day 0 was taken prior to the occupation of the ILMAH system by the student crew, and subsequent samplings were conducted on Day 13, Day 20, and Day 30, the last day of the occupancy. When particulate materials were collected for microbiological examination from cleanroom surfaces, we have shown that at least 1 m^2^ surface area should be sampled so that sufficient biological matter was available to conduct several traditional microbiological and molecular microbial community analyses.

One BiSKit was used to collect samples from one location. The selected area was wiped in three different directions (unidirectional horizontal, vertical, and diagonal) while rotating the sampling device. After the sampling, the dropper attachment handle of the BiSKit was turned to expel the sample, soaked in the macrofoam, into the attached collection bottle. The liquid from the collection bottle was transferred into a sterile 50-ml Falcon tube. To increase sample extraction from each macrofoam, the collection bottle was filled with 15 mL of sterile phosphate-buffered saline (PBS) and reattached to the BiSKit and the macrofoam soaked again in PBS. Then, the dropper attachment was turned one more time to release the sample in the collection tube. The liquid was then transferred into the respective sample collection tube. This step was repeated twice. The sampling liquids (45 mL for each BiSKit) were stored at 4 °C and transported to JPL via overnight courier for further processing. For each sampling time point, a field control (BiSKit open and kept in the air for the time equal to collecting samples) and BiSKit control (unused BiSKit prepared the same way as BiSKits used for sampling) were collected. The protocol for BiSKit sampling and processing was followed as published elsewhere [60]. The time taken to process samples from collection to analyses was within 36 h.

At JPL, the 45-mL liquid samples from each BiSKit were concentrated to ~4-mL samples using the Innovaprep system (INNOVAPEP, Drexel, MO, USA). The concentrated samples were utilized for cultivation and molecular analyses to measure microbial burden and community structure analyses. Briefly, 200 μL of the concentrated sample was mixed with 1.8 mL sterile PBS and thoroughly mixed before performing appropriate dilution(s) and subsequent plating onto suitable agar media and ATP assay. Remaining solutions were used for DNA-based analysis and ~800-μL sampling fluids were stored at 4 °C and used for any contingency.

### Microbiological examination

#### Cultivable microbial examination

For the analysis of cultivable bacterial population, 100 μL of appropriately diluted sample was spread onto two plates of R2A media (Difco). Plates were incubated at room temperature for 7 days. After the cultivation conditions, colony-forming units (CFUs) were counted and reported as CFU/m^2^. The identification and phylogenetic affiliations were carried out via Sanger sequencing targeting the 1–5-kb 16S rRNA gene sequencing. Initially, colony PCR was performed to generate appropriate PCR fragments. However, when the colony PCR step did not yield PCR amplicon, those isolates were subjected to a freeze (−80 °C)–thaw (+80 °C) cycle (suspend one colony in 1-ml PBS, freeze suspension for 15 min, thaw suspension for 15 min, repeat 3 times), automated DNA extraction system [60], and finally, the traditional phenol-chloroform steps [75] to break open cells and enabling to extract DNA. Amplification of the bacterial small-subunit rRNA genes was carried out using primers 8F and 1525R [76]. PCR conditions were as follows: 94 °C for 10 min for denaturation, followed by 35 cycles of 94 °C for 1 min, 55 °C for 1 min, and 72 °C for 1 min 30 s. At the end of the 35 cycles, elongation was carried out at 72 °C for 10 min. The phylogenetic affiliation of the strains was determined by sequencing results with the published type strains sequence database [77] and aligned using ClustalW. Phylogenetic trees were reconstructed using the software MEGA by applying the neighbor-joining method [78].

### Quantitation of total and viable microorganisms

#### ATP assay

Total ATP and intracellular ATP contents representing total and viable microbial population, respectively, were determined using the CheckLite HS kit (Kikkoman, Japan) as previously described [24, 63]. The ATP content was directly correlated with the size of the cells and hence, the ATP values of Gram-positive bacteria were at least 5 times more than Gram-negative cells. Likewise, ~200 times more ATP concentrations were reported in one fungal/yeast-yielding colony compared to 1 CFU of Gram-negative bacteria and spores were devoid of ATP [63]. Similarly, metabolically inactive cells will yield less ATP when compared to the cells that were able to proliferate in favorable conditions [79]. Given these constraints, ATP results were not used to correlate with other bioburden measurements.

#### Sample preparation for molecular assays

The concentrated samples were divided into two aliquots and one of the aliquots was treated with 12.5 μL of PMA (2 mM; Biotium, Inc., Hayward, CA) to a final concentration of 25 μM [80], followed by thorough mixing and incubation in the dark for 5 min at room temperature. Samples were then exposed to light with the PhAST blue-photoactivation system for tubes (GenIUL, S.L., Terrassa, Spain) for 15 min [81]. Information deduced from PMA-treated samples was documented for viable microbial population and data derived from the PMA-untreated aliquot was reported as total (dead and live) microbial population. Both, the PMA-treated and non-treated samples were further split in half, with one half subjected to bead beating with the Fastprep-24 bead-beating instrument (MP Biomedicals, Santa Ana, CA). The samples were run at 5 m/s for 60 s. After bead beating, the samples were combined with their respective analog, which were not subjected to bead beating, and the DNA from the combined sample was extracted by the Maxwell-16 MDx automated system according to the manufacturer’s instructions (Promega, Madison, WI). The purified DNA was eluted into a final volume of 50 μL.

#### qPCR assay

For the analysis of the bacterial and archaeal burden in the samples, real-time quantitative polymerase chain reaction (qPCR) assay, which targets the 16S rRNA gene, was performed in triplicate with a qPCR instrument (BioRad, CFX-96 thermal cycling; Hercules, CA). Universal bacterial primers targeting the 16S gene, 1369F (5′-CGG TGA ATACGT TCY CGG-3′) and modified 1492R (5′-GGW TAC CTTGTT ACG ACT T-3′), and universal archaeal primers targeting the 16S gene, 344af (5′-ACG GGG YGC AGC AGG CGC GA-3′) and 517r (5′-GCC AGC AGC CGC GGT AA-3′), were used [76, 82] to measure bacterial and archaeal burden, respectively. Each 25 μL reaction consisted of 12.5 μL of 2X iQ SYBR Green Supermix (BioRad, Hercules, CA), 1 μL each of forward and reverse oligonucleotide primers, 9.5 μL molecular grade water, and 1 μL of template DNA. The qPCR conditions to determine bacterial burden were: 95 °C; 3 min for denaturation followed by 40 cycles, with each cycle consisting of the following conditions: 10 s hold at 95 °C (denature), 55 °C for primer annealing, and 95 °C for 35 s for extension. Parameters to determine archaeal burden were the following: 95 °C; 15 min for denaturation followed by 40 cycles, with each cycle consisting of the following conditions: 15 s hold at 94 °C, 30 s at 60 °C for annealing, and 30 s at 72 °C for 30 s.

### Molecular microbial diversity analysis

#### Illumina sequencing

The DNA samples were quantified using a Qubit 2.0 fluorometer (Invitrogen, Carlsbad, CA). Bacterial primers 519wF and 1017R were used to amplify a ~500-bp fragment spanning the V4 hypervariable regions of the bacterial 16S rRNA gene [83]. Sequencing was carried out at the Research and Testing Laboratory (Lubbock, TX).

#### Bioinformatic analysis of Illumina sequences

The bacterial and archaeal Illumina-generated MiSeq quality reads were processed and analyzed using the LotuS software [84]. Sequences are screened for quality and discarded if (a) the quality score falls below a threshold of 25, (b) there is one ambiguous base call or more, (c) there is a homonucleotide run in sequence longer than 8, or (d) the final length after trimming and removal of primer sequences and barcodes is lower than 170 bp. Sequences were demultiplexed and clustered into OTUs based on their sequence similarity (97 %) with UPARSE [85]. Taxonomic classification was assigned using the Ribosomal Database Project (RDP) classifier with a confidence of 0.8, and taxonomic abundance was calculated and tabulated.

#### Statistical analyses

An in-house R-script employing the libraries vegan, ape, gplots, mgcv, and GUniFrac was used to compare the bacterial and archaeal Illumina data [31, 86]. Each dataset consisting of the OTU abundances per sample was rarefied 1,000 times to the lowest number of reads and an average Bray-Curtis distance was calculated. This distance was then utilized to calculate non-metric multidimensional scaling (NMDS) or principal coordinate analysis (PCoA), PERMANOVA (Adonis test) and multi-response permutation procedure (MRPP). In addition, the OTU abundances per sample of each dataset were sum-normalized and used to employ either an analysis of variance (ANOVA) or a Spearman rank correlation on the statistical significant changing parameters, and to generate a heat map (*p* value of 0.05). The change of diversity was measured via the Shannon-Wiener diversity index. OTUs that were unclassified at phylum level were removed. When warranted, the *p* value was decreased to only represent high-abundant OTUs and remove false positive results. Heat maps were presented at family level.

### Availability of supporting data

The data set supporting the results of this article is available in the NCBI SRA repository, under accession # SRP069729.

## Additional files

Additional file 1: Statistical analysis. **Tables S1.** Statistical analysis (paired *t* test) to compare the microbial populations of the different time points. **Table S2.** Statistical analysis (paired *t* test) to compare the microbial populations of different locations. **Table S3.** Taxonomic affiliation of cultivable bacterial isolates. **Table S4.** Number of archaeal OTUs associated with ILMAH surfaces collected at various time points. (DOCX 23 kb) Additional file 2: ILMAH architecture, NMDS ordinations based on Bray-Curtis distances, and UniFrac distance of various ILMAH bacteriome datasets. **Figure S1.** Schematic representation of the ILMAH architecture with dimensions. The sampling locations are indicated with stars and numbers (1, 2: bedroom; 3, 4: kitchen; 5: bathroom; and 6, 7, 8: laboratory). **Figure S2.** NMDS ordinations based on Bray-Curtis distances between all samples that were not treated with PMA. (A) All time points, Adonis p-value 0.001, MRPP significance of delta 0.001. (B) all time points except Day 0, Adonis p-value 0.001, MRPP significance of delta 0.001, A: 0.1257. (C) All locations, Adonis p-value 0.493, MRPP significance of delta 0.977, A: –0.09709. (D) all locations except Day 0, Adonis p-value 0.597, MRPP significance of delta 0.413, A: 0.00659. **Figure S3**. NMDS ordinations based on Bray-Curtis distances between all samples that were treated with PMA. (A) All time points, Adonis p-value 0.001, MRPP significance of delta 0.001, A: 0.2813. (B) All time points except Day 0, Adonis p-value 0.001, MRPP significance of delta 0.001, A: 0.06871. (C) All locations, Adonis p-value 0.515, MRPP significance of delta 1, A: –0.09423. (D) all locations except Day 0, Adonis p-value 0.127, MRPP significance of delta 0.905, A: –0.03109. **Figure S4**. UniFrac distance of various ILMAH bacteriome datasets. The bacteriome distribution patterns were similar whether Bray-Curtis distance (A) or UniFrac distance (B) was used. Likewise, significance levels were same. UniFrac for various time points: Adonis *p* value of 0.001, MRPP significance of delta 0.001, A = 0.2988; UniFrac for various locations: Adonis *p* value of 0.801, MRPP significance of delta 0.955, A = −0.03882; variable test: Adonis *p* value of 0.012, MRPP significance of delta 0.004, A = 0.03886). (PDF 527 kb)

## Abbreviations

16S rRNA: small subunit of ribosomal ribose nucleic acid
ANOVA: analysis of variance
ATP: adenosine triphosphate
BiSKit: Biological Sampling Kit
CFU: colony-forming units
DNA: deoxyribose nucleic acid
ILMAH: inflatable lunar/Mars analogous habitat
ISS: International Space Station
MRPP: multi-response permutation procedure
NCBI: National Center for Biotechnology Information
NMDS: non-metric multidimensional scaling
OTU: operational taxonomic unit
PBS: phosphate-buffered saline
PCoA: principal coordinate analysis
PMA: Propidium monoazide
qPCR: quantitative polymerase chain reaction
RDP: Ribosomal Database Project
SRA: Sequence Read Archive
UND: University of North Dakota
US: United States
