# Supplementary material for: Microbial succession in an inflated lunar/Mars analog habitat during a 30-day human occupation
Source: Microbiome. 2016 Jun 2;4:22. doi: 10.1186/s40168-016-0167-0 (PMC4890489; doi:10.1186/s40168-016-0167-0)
Supplement: Additional file 1: — Statistical analysis. Tables S1. Statistical analysis (paired t test) to compare the microbial populations of the different time points. Table S2. Statistical analysis (paired t test) to compare the microbial populations of different locations. Table S3. Taxonomic affiliation of cultivable bacterial isolates. Table S4. Number of archaeal OTUs associated with ILMAH surfaces collected at various time points. (DOCX 23 kb) [file 40168_2016_167_MOESM1_ESM.docx]

| **Table S1. Statistical analysis (paired t-test) to compare the microbial populations of the different time points** | | | | | | |
| --- | --- | --- | --- | --- | --- | --- |
| Biological assay | P value | | | | | |
|  |  |  |  |  |  |  |
|  | 30-0/30-13 | 30-0/30-20 | 30-0/30-30 | 30-13/30-20 | 30-13/30-30 | 30-20/30-30 |
| Total microbes (total ATP) | 0.05* | 0.002* | 0.15 | 0.67 | 0.15 | 0.06 |
| Viable microbes (intracellular ATP) | 0.09 | 0.18 | 0.09 | 0.05* | 0.82 | 0.02* |
| Cultivable bacteria | 0.05* | 0.05* | 0.05* | 0.50 | 0.10 | 0.03* |
| Total bacteria (qPCR) | 0.60 | 0.50 | 0.15 | 0.10 | 0.55 | 0.72 |
| Viable bacteria (PMA-qPCR) | 0.15 | 0.28 | 0.38 | 0.02* | 0.21 | 0.66 |

| **Table S2. Statistical analysis (paired t-test) to compare the microbial populations of different locations** | | | | | | | |
| --- | --- | --- | --- | --- | --- | --- | --- |
| Biological assay | | | P-value | | | | |
|  |  |  |  |  |  |  |  |
|  |  |  | Bedroom/Kitchen | | Bedroom/Bathroom | | Bedroom/Lab |
| Total microbes (total ATP) | | | 0.70 |  | 0.23 |  | 0.71 |
| Viable microbes (intracellular ATP) | | | 0.37 |  | 0.38 |  | 0.24 |
| Cultivable bacteria | | | 0.43 |  | 0.09 |  | 0.40 |
| Total bacteria (qPCR) | | | 0.19 |  | 0.42 |  | 0.21 |
| Viable bacteria (PMA-qPCR) | | | 0.18 |  | 0.50 |  | 0.39 |
|  |  |  |  |  |  |  |  |
|  |  |  | Kitchen/Bathroom |  | Kitchen/Lab |  | Bathroom/Lab |
| Total microbes (total ATP) | | | 0.20 |  | 0.89 |  | 0.19 |
| Viable microbes (intracellular ATP) | | | 0.51 |  | 0.29 |  | 0.32 |
| Cultivable bacteria | | | 0.40 |  | 0.39 |  | 0.39 |
| Total bacteria (qPCR) | | | 0.70 |  | 0.32 |  | 0.78 |
| Viable bacteria (PMA-qPCR) | | | 0.93 |  | 0.94 |  | 0.99 |

**Table S3. Taxonomic affiliation of cultivable bacterial isolates**

| Strain # | Strain identity EZ taxon | % similarity with type strain | Accession # EZ | Phyla |
| --- | --- | --- | --- | --- |
| B01-01 | *Achromobacter ruhlandii* | 97.75 | AB010840 | betaproteobacteria |
| B01-02 | *Curtobacterium plantarum* | 93.91 | JN175348 | Actinobacteria |
| B01-03 | *Bacillus idriensis* | 99.93 | AY904033 | Firmicutes |
| B01-04 | *Prolinoborus fasciculus CIP 103579(T)* | 98.99 | JN175353 | Betaproteobacteria |
| B01-05 | *Pseudomonas mandelii* | 99.43 | AF058286 | Gammaproteobacteria |
| B02-01 | *Bacillus litoralis SW-211(T)* | 98.1 | AY608605 | Firmicutes |
| B02-02 | *Bacillus circulans* | 99.16 | AY724690 | Firmicutes |
| B02-03 | *Pseudomonas graminis* | 99.93 | Y11150 | Gammaproteobacteria |
| B02-04 | *Pseudomonas graminis* | 99.93 | Y11150 | Gammaproteobacteria |
| B02-05 | *Sphingomonas aerolata* | 99.7 | AJ429240 | Alphaproteobacteria |
| B03-01 | *Bacillus niacini* | 99.37 | AB021194 | Firmicutes |
| B03-02 | *Bacillus korlensis* | 98.51 | EU603328 | Firmicutes |
| B03-03 | *Bacillus idriensis* | 99.86 | AY904033 | Firmicutes |
| B03-04 | *Sphingomonas panni* | 99.56 | AJ575818 | Alphaproteobacteria |
| B03-05 | *Pedobacter duraquae* | 98.4 | [AM491368](javascript:%20reloadBrowse('AM491368',%20'www.ezbiocloud.net');) | Bacteroidetes |
| B04-01 | *Pseudomonas graminis* | 99.86 | Y11150 | Gammaproteobacteria |
| B04-02 | *Paenibacillus agarexedens* | 98.8 | AJ345020 | Firmicutes |
| B04-03 | *Pseudomonas graminis* | 99.79 | Y11150 | Gammaproteobacteria |
| B04-04 | *Sphingomonas aerolata* | 99.7 | AJ429240 | Alphaproteobacteria |
| B04-05 | *Bacillus beringensis BR035(T)* | 98.87 | FJ889576 | Firmicutes |
| B05-01 | *Stenotrophomonas rhizophila* | 99.79 | CP007597 | Gammaproteobacteria |
| B05-02 | *Exiguobacterium sibiricum 255-15(T)* | 99.04 | CP001022 | Firmicutes |
| B05-03 | *Frigoribacterium faeni* | 99.35 | Y18807 | Actinobacteria |
| B05-04 | *Bacillus idriensis* | 99.86 | AY904033 | Firmicutes |
| B05-05 | *Bacillus idriensis* | 99.86 | AY904033 | Firmicutes |
| B06-01 | *Massilia aurea AP13(T)* | 98.64 | AM231588 | Betaproteobacteria |
| B06-02 | *Massilia timonae* | 97.93 | AGZI01000009 | Betaproteobacteria |
| B06-03 | *Pseudomonas koreensis* | 99.72 | AF468452 | Gammaproteobacteria |
| B06-04 | *Pseudomonas ficuserectae JCM 2400(T)* | 97.08 | AB021378 | Gammaproteobacteria |
| B06-05 | *Sphingomonas aerolata* | 99.55 | AJ429240 | Alphaproteobacteria |
| B07-01 | *Clavibacter michiganensis subsp tessellarius* | 100 | U30254 | Actinobacteria |
| B07-02 | *Pseudomonas koreensis* | 99.64 | AF468452 | Gammaproteobacteria |
| B07-03 | *Duganella zoogloeoides* | 99.64 | [D14256](javascript:%20reloadBrowse('D14256',%20'www.ezbiocloud.net');) | Betaproteobacteria |
| B07-04 | *Pseudomonas mandelii* | 99.43 | AF058286 | Gammaproteobacteria |
| B07-05 | *Pseudomonas koreensis* | 99.5 | AF468452 | Gammaproteobacteria |
| B08-01 | *Pseudomonas chlororaphis subsp. Piscium JF3835(T)* | 97.82 | FJ168539 | Gammaproteobacteria |
| B08-02 | *Pseudomonas meridiana* | 99.5 | AJ537602 | Gammaproteobacteria |
| B08-03 | *Pseudomonas seleniipraecipitans CA5(T)* | 99.24 | FJ422810 | Gammaproteobacteria |
| B08-04 | *Pseudomonas veronii* | 99.86 | AF064460 | Gammaproteobacteria |
| B08-05 | *Pseudomonas seleniipraecipitans CA5(T)* | 99.32 | FJ422810 | Gammaproteobacteria |
| B21-01 | *Methylobacterium marchantiae* | 99.78 | FJ157976 | Alphaproteobacteria |
| B21-02 | *Bacillus megaterium* | 99.78 | D16273 | Firmicutes |
| B21-03 | *Staphylococcus aureus subsp. Anaerobius* | 99.86 | D83355 | Firmicutes |
| B21-04 | *Staphylococcus epidermis* | 100 | L37605 | Firmicutes |
| B22-01 | *Bacillus aryabhattai* | 99.86 | EF114313 | Firmicutes |
| B22-02 | *Microvirga aerilata 5420S-16(T)* | 96.87 | GQ421849 | Alphaproteobacteria |
| B22-03 | *Microbacterium phylloshpaerae* | 99.06 | AJ277840 | Actinobacteria |
| B22-04 | *Staphylococcus aureus subsp. Anaerobius* | 99.65 | D83355 | Firmicutes |
| B22-05 | *Staphylococcus aureus subsp. Anaerobius* | 99.86 |  | Firmicutes |
| B23-01 | *Rhodococcus rhodochrous* | 99.93 | X79288 | Actinobacteria |
| B23-02 | *Anoxybacillus suryakundensis JS1(T)* | 99.23 | KC958552 | Firmicutes |
| B23-03 | *no sequence* |  |  |  |
| B23-04 | *Bacillus pumilus* | 99.86 | ABRX01000007 | Firmicutes |
| B23-05 | *Microbacterium yannicii* | 98.99 | FN547412 | Actinobacteria |
| B24-01 | *Bacillus niacicni IFO 15566(T)* | 99.51 | AB021194 | Firmicutes |
| B24-02 | *Bacillus idriensis* | 99.86 | AY904033 | Firmicutes |
| B24-03 | *Sphingomonas faeni* | 97.83 | AJ429239 | Alphaproteobacteria |
| B24-04 | *no sequence* |  |  |  |
| B24-05 | *Micrococcus yunnanensis* | 99.57 | FJ214355 | Actinobacteria |
| B25-01 | *Paenibacillus tundrae* | 99.93 | EU558284 | Firmicutes |
| B25-02 | *Microbacterium invictum* | 98.62 | AM949677 | Actinobacteria |
| B25-03 | *Paenibacillus agarexedens DSM 1327(T)* | 98.66 | AJ345020 | Firmicutes |
| B25-03 | *Staphylococcus lugdunensis* | 100 | AB009941 | Firmicutes |
| B25-04 | *Staphylococcus lugdunensis* | 99.86 | AB009941 | Firmicutes |
| B25-05 | *Rhodococcus rhodochrous* | 99.86 | X79288 | Actinobacteria |
| B26-01 | *Achromobacter marplatensis* | 99.71 | EU150134 | Betaproteobacteria |
| B26-02 | *Bacillus soli LMG 21838(T)* | 97.8 | AJ542513 | Firmicutes |
| B26-03 | *Bacillus niacini* | 99.37 | AB021194 | Firmicutes |
| B26-04 | *Bacillus idriensis* | 99.93 | AY904033 | Firmicutes |
| B26-05 | *Bacillus litoralis SW-211(T)* | 98.11 | AY608605 | Firmicutes |
| B27-01 | *Bacillus siamensis* | 99.93 | AJVF01000043 | Firmicutes |
| B27-02 | *Curtobacterium flaccumfaciens LMG 3645(T)* | 100 | AJ312209 | Actinobacteria |
| B27-03 | *Bacillus idriensis* | 99.65 | AY904033 | Firmicutes |
| B27-04 | *Staphylococcus aureus subsp. Anaerobius* | 99.93 | D83355 | Firmicutes |
| B27-05 | *Anoxybacillus suryakundensis JS1(T)* | 99.23 | KC958552 | Firmicutes |
| B28-01 | *Curtobacterium flaccumfaciens LMG 3645(T)* | 100 | AJ312209 | Actinobacteria |
| B28-02 | *Bacillus litoralis* | 99.86 | AY608605 | Firmicutes |
| B28-03 | *Bacillus niacini* | 99.51 | AB021194 | Firmicutes |
| B28-04 | *Curtobacterium citreum DSM 20528(T)* | 98.85 | X77436 | Actinobacteria |
| B28-05 | *Nocardioides ganghwensis JC2055 (T)* | 98.48 | AY423718 | Actinobacteria |
| B31-01 | *Bacillus niacini* | 99.37 | AB021194 | Firmicutes |
| B31-02 | *Staphylococcus hominis subsp. Hominis* | 99.86 | X66101 | Firmicutes |
| B31-03 | *Bacillus aryabhattai* | 100 | EF114313 | Firmicutes |
| B31-04 | *Bacillus idriensis* | 99.93 | AY904033 | Firmicutes |
| B31-05 | *Curtobacterium flaccumfaciens* | 99.04 | AJ312209 | Actinobacteria |
| B32-01 | *Sphingomonas mucosissima CP173-2(T)* | 98.67 | AM229669 | Alphaproteobacteria |
| B32-02 | *Kocuria rosea DSM 2044(T)* | 99.86 | X87756 | Actinobacteria |
| B32-03 | *Bacillus megaterium* | 99.79 | D16273 | Firmicutes |
| B32-04 | *Anoxybacillus suryakundensis JS1(T)* | 99.09 | KC958552 | Firmicutes |
| B32-05 | *Bacillus subtilis subsp. Inaquosorum* | 99.93 | AMXN01000021 | Firmicutes |
| B33-01 | *Bacillus gottheilii WCC 4585(T)* | 98.17 | FN995266 | Firmicutes |
| B33-02 | *Bacillus aryabhattai* | 99.86 | EF114313 | Firmicutes |
| B33-03 | *Paenibacillus agarexedens (T)* | 93.79 | AJ345020 | Firmicutes |
| B33-04 | *Bacillus aryabhattai* | 100 | EF114313 | Firmicutes |
| B33-05 | *Staphylococcus aureus subsp. Anaerobius* | 99.93 | D83355 | Firmicutes |
| B34-01 | *Microbacterium arthrosphaerae CC-VM_Y(T)* | 98.92 | FN870023 | Actinobacteria |
| B34-02 | *Staphylococcus hominis subsp. Hominis* | 99.65 | X66101 | Firmicutes |
| B34-03 | *Staphylococcus hominis subsp. Hominis* | 99.65 | X66101 | Firmicutes |
| B34-04 | *Staphylococcus aureus subsp. Anaerobius* | 99.93 | D83355 | Firmicutes |
| B35-01 | *Microbacterium aerolatum V-73(T)* | 99.7 | AJ309929 | Actinobacteria |
| B35-02 | *Staphylococcus aureus subsp. Anaerobius* | 99.93 | D83355 | Firmicutes |
| B35-03 | *Microbacterium invictum* | 98.71 | [AM949677](javascript:%20reloadBrowse('AM949677',%20'www.ezbiocloud.net');) | Actinobacteria |
| B35-04 | *Bacillus funiculus* | 99.65 | AB049195 | Firmicutes |
| B35-05 | *Sphingomonas paucimobilis* | 99.84 | U20776 | Alphaproteobacteria |
| B36-01 | *Bacillus idriensis* | 99.86 | [AY904033](javascript:%20reloadBrowse('AY904033',%20'www.ezbiocloud.net');) | Firmicutes |
| B36-02 | *Paenisporosarcina quisquiliarum* | 99.02 | DQ333897 | Firmicutes |
| B36-03 | *Bacillus niacini IFO 15566 (T)* | 98.81 | AB021194 | Firmicutes |
| B36-04 | *Staphylococcus saprophyticus subsp. Bovis* | 100 | AB233327 | Firmicutes |
| B36-05 | *Frigoribacterium faeni 801(T)* | 97.81 | Y18807 | Actinobacteria |
| B37-01 | *Bacillus boroniphilus* | 99.79 | BAUW010000204 | Firmicutes |
| B37-02 | *Paenibacillus tundrae* | 99.93 | EU558284 | Firmicutes |
| B37-03 | *Bacillus beringensis* | 96.87 | FJ889576 | Firmicutes |
| B37-04 | *Bacillus halmapalus* | 98.57 |  | Firmicutes |
| B38-01 | *Frigoribacterium faeni* | 99.63 | Y18807 | Actinobacteria |
| B38-02 | *Rhodococcus wratislaviensis* | 100 | [AJ786666.1](http://www.ebi.ac.uk/ena/data/view/AJ786666.1) | Actinobacteria |
| B38-03 | *Bacillus idriensis* | 99.86 | AY904033 | Firmicutes |
| B38-04 | *Anoxybacillus suryakundensis JS1(T)* | 99.23 | KC958552 | Firmicutes |
| B41-01 | *Staphylococcus aureus subsp. Anaerobius* | 99.93 | D83355 | Firmicutes |
| B41-02 | *Staphylococcus aureus subsp. Anaerobius* | 99.79 | D83355 | Firmicutes |
| B41-03 | *Brevundimonas bullata* | 99.63 | D12785 | Alphaproteobacteria |
| B41-04 | *Brevundimonas bullata* | 99.77 |  | Alphaproteobacteria |
| B41-05 | *Streptomyces collinus* | 99.86 | AB184123 | Actinobacteria |
| B42-01 | *Sphingomonas aerolata* | 99.78 | AJ429240 | Alphaproteobacteria |
| B42-02 | *Microbacterium testaceum* | 99.35 | X77445 | Actinobacteria |
| B42-03 | *Frigoribacterium faeni* | 99.64 | Y18807 | Actinobacteria |
| B42-04 | *Pseudomonas koreensis* | 99.79 | AF468452 | Gammaproteobacteria |
| B42-05 | *Arthrobacter agilis* | 99.71 | X80748 | Actinobacteria |
| B43-01 | *Curtobacterium luteum* | 100 | X77437 | Actinobacteria |
| B43-02 | *Paenibacillus tundrae* | 99.93 | EU558284 | Firmicutes |
| B43-03 | *Staphylococcus warneri* | 99.86 | L37603 | Firmicutes |
| B45-01 | *Bacillus idriensis* | 99.79 | AY904033 | Firmicutes |
| B45-02 | *Bacillus aryabhattai* | 99.93 | EF114313 | Firmicutes |
| B45-03 | *Staphylococcus hominis subsp. Hominis* | 99.86 | X66101 | Firmicutes |
| B45-04 | *Bacillus aryabhattai* | 100 | EF114313 | Firmicutes |
| B45-05 | *Staphylococcus aureus subsp. Anaerobius* | 99.79 | D83355 | Firmicutes |
| B46-01 | *Bacillus safensis* | 99.93 | ASJD01000027 | Firmicutes |
| B46-02 | *Staphylococcus lugdunensis ATCC 43809(T)* | 99 | AB009941 | Firmicutes |
| B46-03 | *Sphingomonas yunnanensis* | 99.78 | AY894691 | Alphaproteobacteria |
| B46-04 | *Frigoribacterium faeni* | 99.64 | Y18807 | Actinobacteria |
| B46-05 | *Staphylococcus cohnii subsp. Cohnii* | 100 | D83361 | Firmicutes |
| B47-01 | *Bacillus halmapalus* | 99.15 | X76447 | Firmicutes |
| B47-02 | *Frigoribacterium faeni* | 99.27 | Y18807 | Actinobacteria |
| B47-03 | *Staphylococcus aureus subsp. Anaerobius* | 99.93 | D83355 | Firmicutes |
| B47-04 | *Streptomyces thermocarboxydus* | 99.93 | U94490 | Actinobacteria |
| B47-05 | *Staphylococcus aureus subsp. Anaerobius* | 99.86 | D83355 | Firmicutes |
| B48-01 | *Sphingomonas aquatilis* | 99.41 | AF131295 | Alphaproteobacteria |
| B48-02 | *Pseudomonas koreensis* | 99.86 | AF468452 | Gammaproteobacteria |
| B48-03 | *Flavobacterium oceanosedimentum ATCC31317* | 99.21 | EF592577 | Bacteroidetes |
| B48-04 | *Staphylococcus saprophyticus subsp. Bovis* | 100 | AB233327 | Firmicutes |
| B48-05 | *Paenibacillus agarexedens* | 98.66 | AJ345020 | Firmicutes |

**Table S4. Number of archaeal OTUs associated with ILMAH surfaces collected at various time points.**

| Archaeal taxon | Number of archaeal OTUs at timepoint: | | | | | | | | | | |  |
| --- | --- | --- | --- | --- | --- | --- | --- | --- | --- | --- | --- | --- |
|  | Day-0 | | Day-13 | | | Day-20 | | | Day-30 | | |  |
|  | No PMA | PMA | | No PMA | PMA | | No PMA | PMA | | No PMA | PMA | |
| Methanocaldococcaceae (E) |  |  | | 119 |  | |  |  | |  |  | |
| Methanosarcinaceae (E) |  |  | | 2 |  | |  |  | | 66 |  | |
| Nitrosopumilaceae (T) |  |  | | 2690 |  | |  |  | | 322 |  | |
| Nitrososphaeraceae (T) | 15010 | 27 | | 14988 | 53 | | 24 |  | | 13446 | 15 | |
