# Supplementary figures and images for: Microbial succession in an inflated lunar/Mars analog habitat during a 30-day human occupation
Source: Microbiome. 2016 Jun 2;4:22. doi: 10.1186/s40168-016-0167-0 (PMC4890489; doi:10.1186/s40168-016-0167-0)

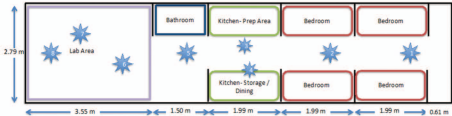

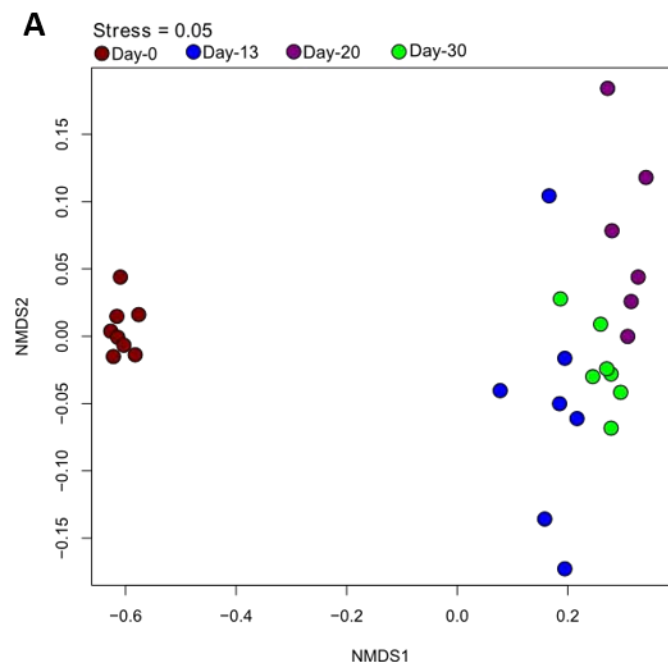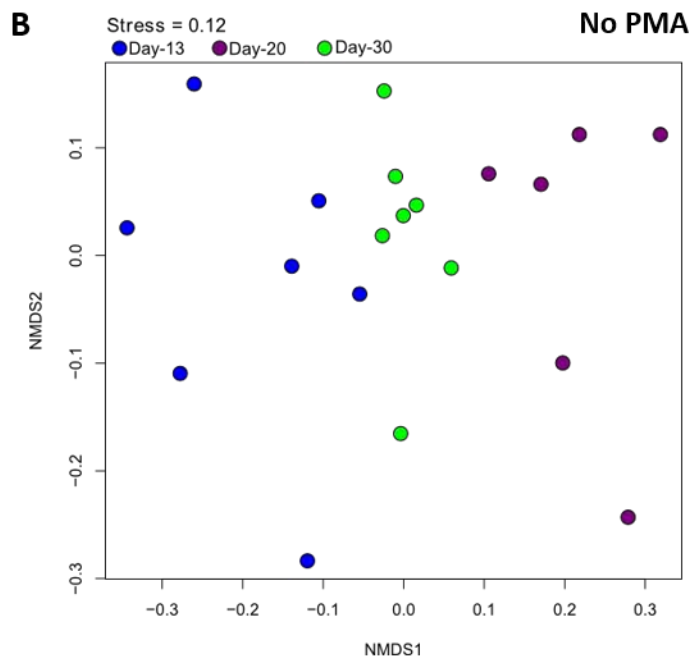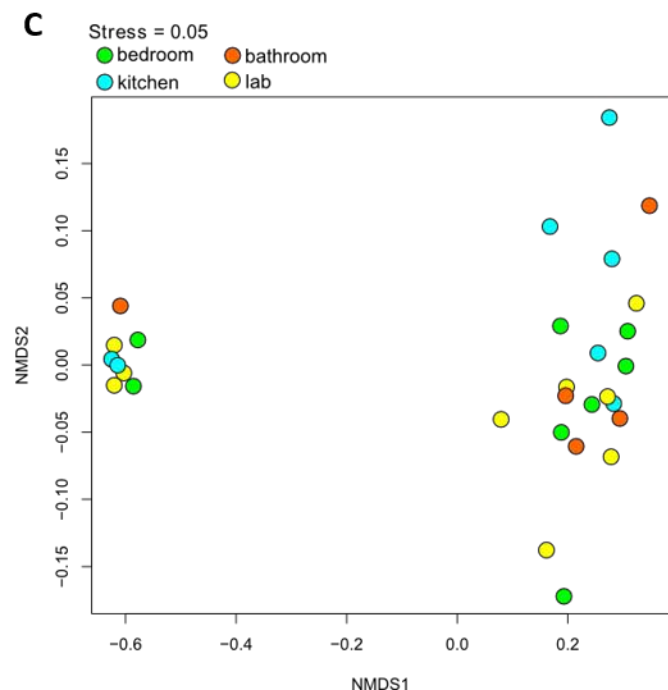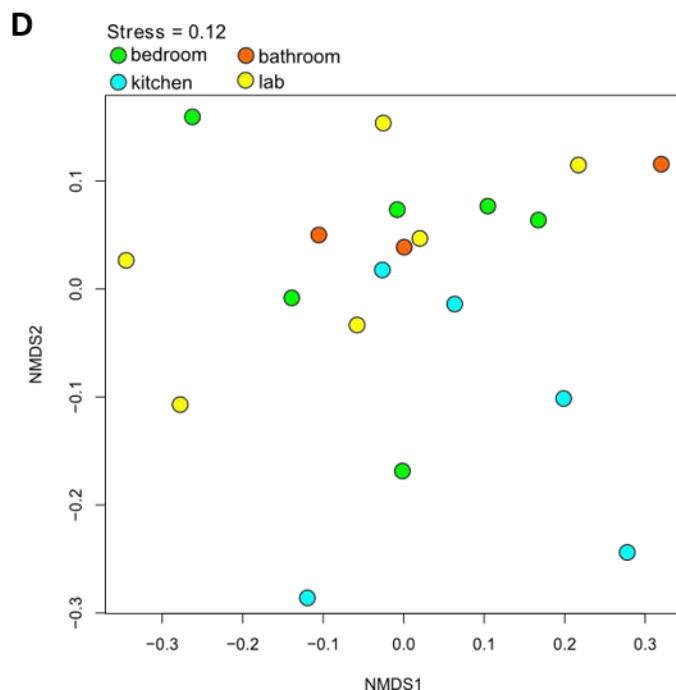

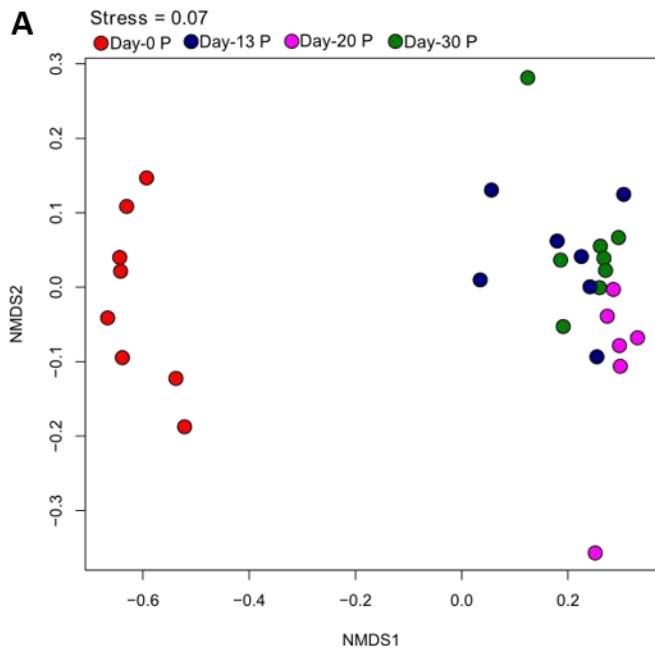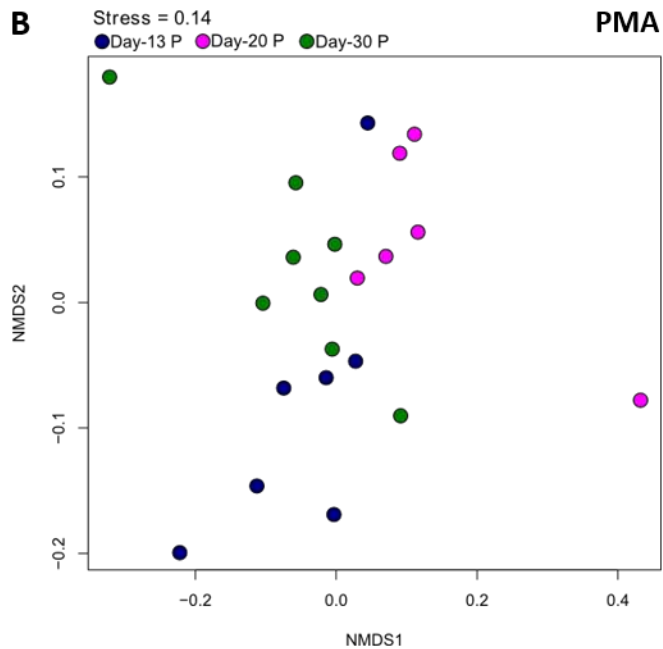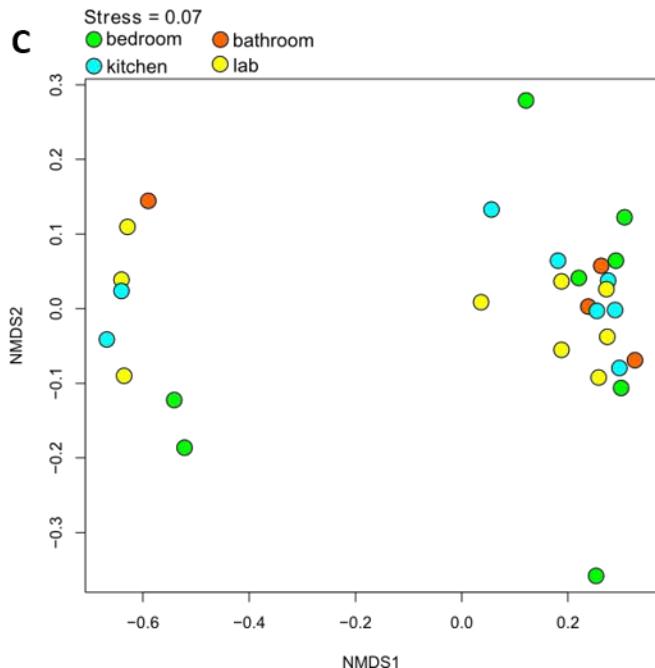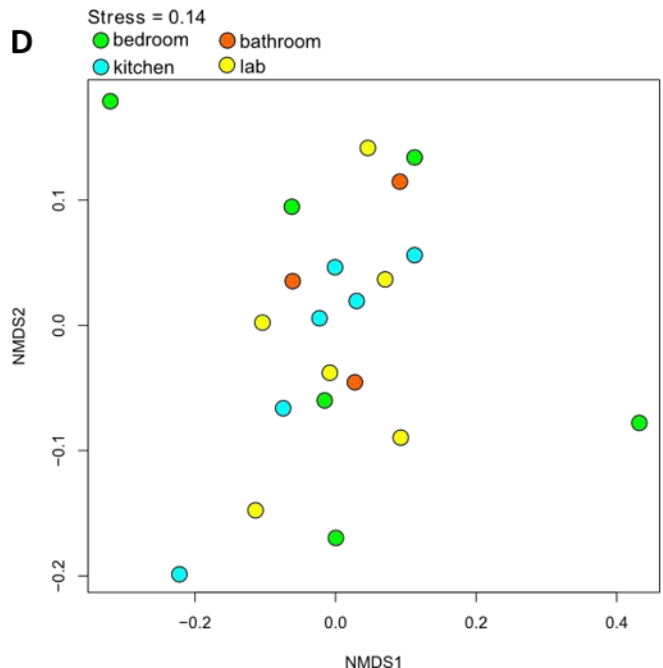

**A**

Stress = 0.09

Day-0 Day-13 Day-20 Day-30  
Day-0 P Day-13 P Day-20 P Day-30 P

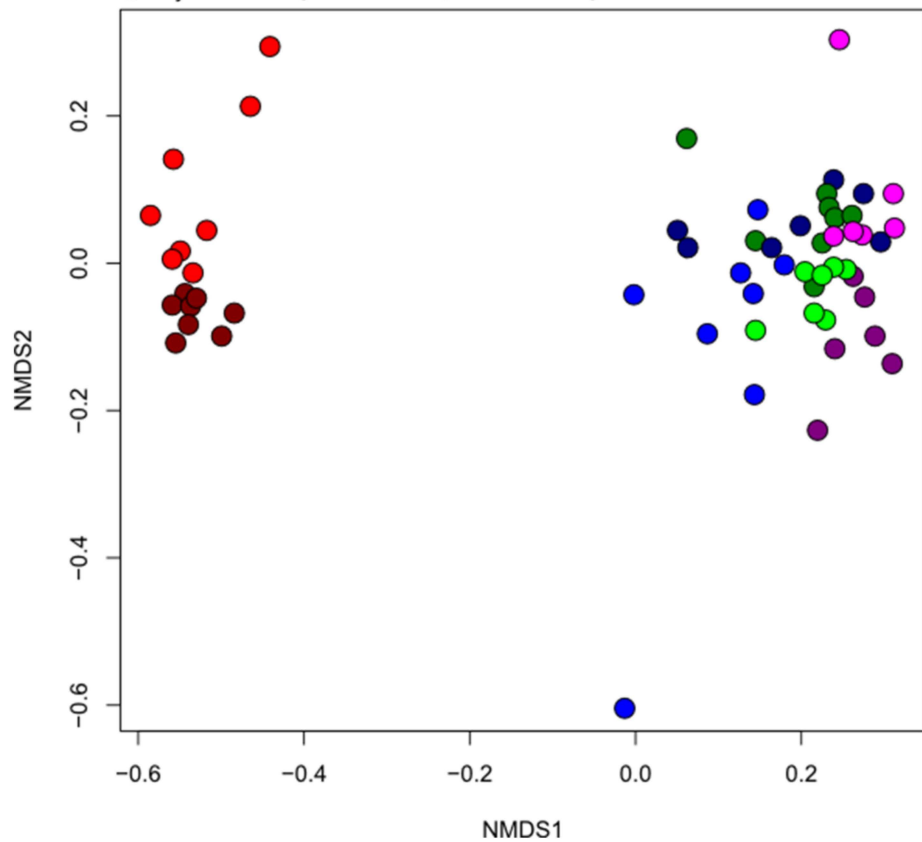**B**

Stress = 0.08

Day-0 Day-13 Day-20 Day-30  
Day-0 P Day-13 P Day-20 P Day-30 P

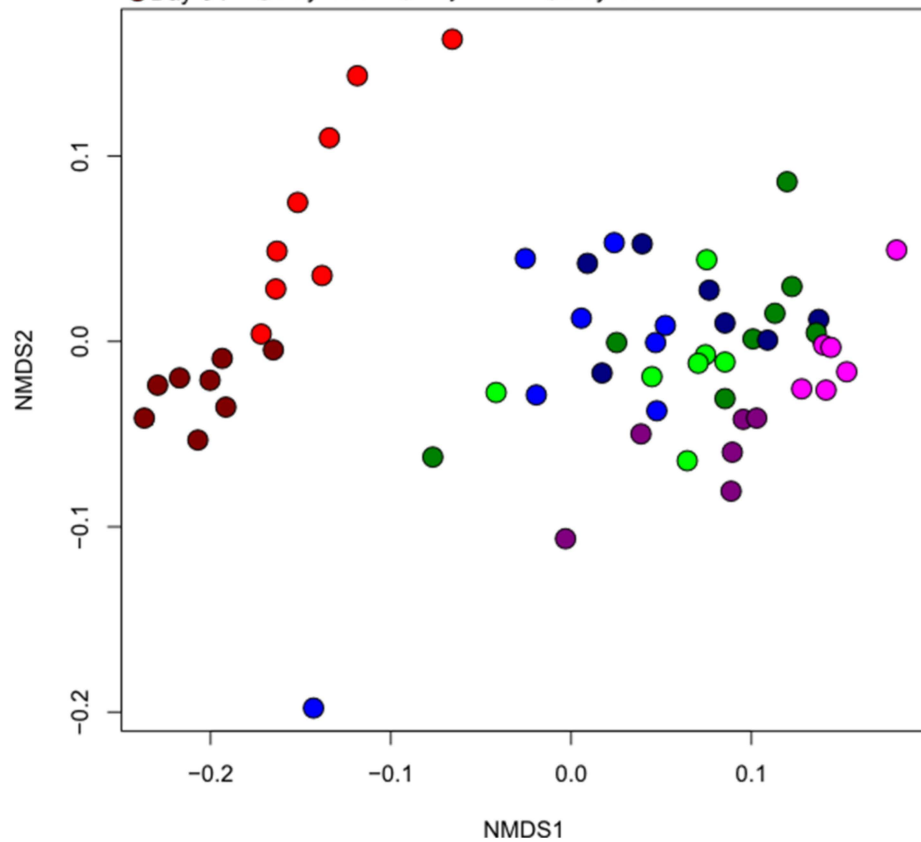

Supplement: Additional file 2: — ILMAH architecture, NMDS ordinations based on Bray-Curtis distances, and UniFrac distance of various ILMAH bacteriome datasets. Figure S1. Schematic representation of the ILMAH architecture with dimensions. The sampling locations are indicated with stars and numbers (1, 2: bedroom; 3, 4: kitchen; 5: bathroom; and 6, 7, 8: laboratory). Figure S2. NMDS ordinations based on Bray-Curtis distances between all samples that were not treated with PMA. (A) All time points, Adonis p-value 0.001, MRPP significance of delta 0.001. (B) all time points except Day 0, Adonis p-value 0.001, MRPP significance of delta 0.001, A: 0.1257. (C) All locations, Adonis p-value 0.493, MRPP significance of delta 0.977, A: –0.09709. (D) all locations except Day 0, Adonis p-value 0.597, MRPP significance of delta 0.413, A: 0.00659. Figure S3. NMDS ordinations based on Bray-Curtis distances between all samples that were treated with PMA. (A) All time points, Adonis p-value 0.001, MRPP significance of delta 0.001, A: 0.2813. (B) All time points except Day 0, Adonis p-value 0.001, MRPP significance of delta 0.001, A: 0.06871. (C) All locations, Adonis p-value 0.515, MRPP significance of delta 1, A: –0.09423. (D) all locations except Day 0, Adonis p-value 0.127, MRPP significance of delta 0.905, A: –0.03109. Figure S4. UniFrac distance of various ILMAH bacteriome datasets. The bacteriome distribution patterns were similar whether Bray-Curtis distance (A) or UniFrac distance (B) was used. Likewise, significance levels were same. UniFrac for various time points: Adonis p value of 0.001, MRPP significance of delta 0.001, A = 0.2988; UniFrac for various locations: Adonis p value of 0.801, MRPP significance of delta 0.955, A = −0.03882; variable test: Adonis p value of 0.012, MRPP significance of delta 0.004, A = 0.03886). (PDF 527 kb) [file 40168_2016_167_MOESM2_ESM.pdf]
